# Interpersonal Affective Touch in a Virtual World: Feeling the Social Presence of Others to Overcome Loneliness

**DOI:** 10.3389/fpsyg.2021.795283

**Published:** 2022-01-11

**Authors:** Letizia Della Longa, Irene Valori, Teresa Farroni

**Affiliations:** Department of Developmental Psychology and Socialization, University of Padova, Padua, Italy

**Keywords:** interpersonal affective touch, loneliness, virtual reality, autism, anorexia nervosa, interpersonal violence

## Abstract

Humans are by nature social beings tuned to communicate and interact from the very beginning of their lives. The sense of touch represents the most direct and intimate channel of communication and a powerful means of connection between the self and the others. In our digital age, the development and diffusion of internet-based technologies and virtual environments offer new opportunities of communication overcoming physical distance. It however, happens that social interactions are often mediated, and the tactile aspects of communication are overlooked, thus diminishing the feeling of social presence, which may contribute to an increased sense of social disconnection and loneliness. The current manuscript aims to review the extant literature about the socio-affective dimension of touch and current advancements in interactive virtual environments in order to provide a new perspective on multisensory virtual communication. Specifically, we suggest that interpersonal affective touch might critically impact virtual social exchanges, promoting a sense of co-presence and social connection between individuals, possibly overcoming feelings of sensory loneliness. This topic of investigation will be of crucial relevance from a theoretical perspective aiming to understand how we integrate multisensory signals in processing and making sense of interpersonal exchanges, this is important in both typical and atypical populations. Moreover, it will pave the way to promising applications by exploring the possibility to use technical innovations to communicate more interactively in the case of people who suffer from social isolation and disconnection from others.

## Introduction

Human beings constantly seek to stay one close to another and interact suggesting the need to feel connected and establish emotional bonds with others to create and maintain interpersonal relationships (Baumeister and Leary, 1995), which have the potential to shape biological responses and behaviors that are consequential for health and psychological well-being (Pietromonaco and Collins, 2017). Physical contact, mediated by the sense of touch, is an essential part of social communication providing the experience of actual togetherness, which can be defined as social presence (van Erp and Toet, 2015). In case of prolonged periods of social distancing and isolation, such as during the COVID-19 pandemic when physical distancing has been prescribed in order to limit the diffusion of infections, deprivation of interpersonal touch has been associated with greater loneliness and anxiety and people showed to crave intimate tactile interactions, underlining the importance of feeling the physical presence of others for psychological well-being (Banerjee et al., 2021; von Mohr et al., 2021). Like never before, social distancing measures have been globally imposed, offering a new lens through which the psychosocial impact of deprivation of tactile interactions should be examined. This reevaluation of the importance of physical presence and interpersonal touch is particularly important considering an increasing use of virtual environments that strongly rely on vision and audition, but scarcely involve tactile stimulations. Indeed, current communication systems, such as videoconferencing, social media use and engagement with virtual reality activities, do not support sensory feedback through the sense of touch and they have been shown to be not sufficient to prevent social isolation and loneliness (Usta et al., 2014; Twenge et al., 2019; Boursier et al., 2020). More specifically, people reported to spend more time on social media and virtual reality activities during prolonged periods of isolation and physical distancing, which helped users keep themselves occupied and active (Siani and Marley, 2021). Feelings of loneliness have been shown to predict time spent using social media that probably strengthened the need to be part of a virtual community; however, the facilitated and prolonged access to Internet and social media risked further increasing anxiety, generating a vicious cycle between loneliness and excessive social media use, that in some cases may require clinical attention (Boursier et al., 2020). In this regard, social media use highlights both opportunities for individuals to face isolation through virtual communication and risky behaviors, depending on its specific use or misuse (Livingstone, 2008). Especially among young people, higher feelings of loneliness have been shown to predict an increased social media use to keep in touch with peers and family, however it was not associated with happiness (Cauberghe et al., 2021). Considering the extensive diffusion of digital technologies, an increasing amount of social interaction is now mediated by communication devices, substituting direct physical contact (Twenge et al., 2019). Virtual exchanges have the advantages of allowing communication between people physically distant, providing a feeling of social presence, named the perception of being present with others within an environment mediated by communication technologies (Triberti et al., 2018). However, if such a perception does not manifest, it may result in an increasing sense of loneliness and social disconnection between people. This suggests that now is an important time to begin intervention efforts targeting especially those people at risk for feeling lonely and unable to connect with others. In this perspective, the development of multisensory virtual environments may also represent an innovative tool for assessing and training social abilities and the sense of social connection, focusing on the role of interpersonal touch in enriching mediated exchanges with crucial social and affective information.

**FIGURE 1:**
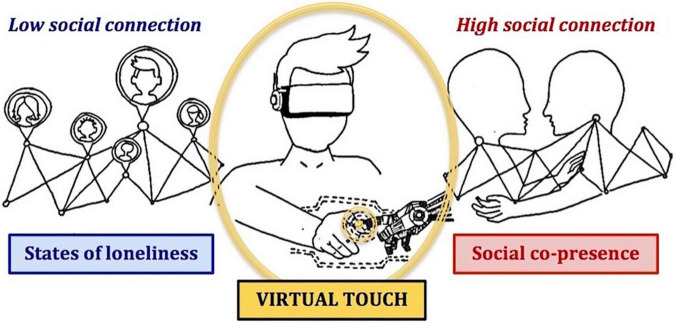
Schematic representation of an integrative perspective on the applicability of interpersonal affective touch in virtual reality (VR) as a potential driver of self-other connection.

The present narrative review aims to consider interpersonal affective touch as an essential component of perceiving social presence during virtual interactions, which may critically modulate the sense of social connection and prevent loneliness. By reviewing the current literature about the social function of tactile stimulation and the advanced opportunities of social interactions in virtual environments, we propose an integrative perspective on the applicability of interpersonal affective touch in VR as a potential driver of self-other connection (Figure 1).

In the first section, we will describe the neurophysiological properties of interpersonal affective touch that support self exploration and social exchanges, specifically assessing the implications of tactile stimulation in modulating feelings of loneliness. In the second section, we will critically examine whether interactive technologies play a role in contributing to or mitigating loneliness. Importantly, we will discuss current challenges in advancing virtual reality by including other sensory channels that may represent new opportunities for communicating socio-affective significance and increasing the feeling of social presence and connection between people who are physically apart. Finally, in the last section, we will explore the most promising technical perspectives to support more interactive communication in the case of people who suffer from social isolation and disconnection from others.

In order to search and select relevant contributions to the body of knowledge of the current review, we combined three strings referring to the main topics of the review, with three additional strings related to the example cases reported in the second part of the manuscript. Thus, we defined six different strings, one for each of the key-words of this manuscript (Table 1). Given the fact that we aimed to link different topics of research that have been rarely studied all together, we used all possible pair combinations of the aforementioned strings in order to provide a broad understanding of the state of the art (e.g., affective touch and loneliness; affective touch and virtual reality; virtual reality and loneliness). Moreover, as we intended to select the contributions focusing on the social connection and virtual communication between individuals, we also included the keywords “Social connection” and “Virtual communication” in our search. In the first step, we searched for references listed in PsycINFO and PubMed. In a second step, we searched reference lists of articles identified by this first search and selected those describing studies that included a specific focus on the topics described above. For the detailed description of the most relevant research studies on which we based this review, see the Table reported in Supplementary Materials. Several review articles and book chapters were also included.

**TABLE 1:** String used to search and select relevant contributions of this review.

	Topic	String
Main topics	Touch	Affective touch OR social touch OR interpersonal touch
	Loneliness	Loneliness OR social isolation OR social exclusion OR lonely
	Virtual reality	Virtual reality OR immersive virtual reality OR VR OR IVR OR Head Mounted Display OR HMD
Example cases	Autism	Autism OR ASD OR autism spectrum disorder OR autistic disorder
	Anorexia	Anorexia nervosa OR anorexia OR anorexic OR eating disorder
	Interpersonal violence	interpersonal violence OR ipv OR aggressive behavior

## Social Connection Through Tactile Experiences

Early sensory experiences and interaction features, mediated by physical contact and interpersonal touch, provide the neuro-behavioral mechanisms supporting the development of social connections and affective bonds (Dunbar, 2008; Su and Su, 2018). Shared sensory experiences may promote the development of predictive internal models concerning others’ affective states and behaviors, critically shaping the ability to feel close and connected with others during interpersonal exchanges (Maister et al., 2015). In particular, interpersonal affective touch, which refers to the emotional and motivational facets of tactile exchanges between social partners, has been shown to modulate psychological boundaries between the self and the others (Gallace and Spence, 2014), thus critically impacting the feeling of social connection. A unique characteristic of tactile interactions is the fact that touch is reciprocal in nature, as it consists in a shared sensory experience between individuals, and so it represents a privileged channel of communication that can convey immediate socio-emotional meanings and reinforce social bonds (Morrison et al., 2010). The dynamics of interpersonal touch rely on different mechanisms and various levels: sensory properties of tactile stimulation, physiological responses (including changes in hormone levels), and emotional experiences (Cascio et al., 2019). All these processes interact and possibly reinforce each other, thus providing the complex sensation of feeling in touch and connected with another person.

### Neurophysiology of Interpersonal Affective Touch: A Bridge Between the Self and the Others

The skin is the largest sensory organ surrounding the whole human body and it is innervated by a wide array of sensory fibers supporting the transduction and processing of thermo-mechanical stimulation on the body surface (McGlone and Reilly, 2010). This strategic position makes the skin an important point of interchanges between the body and the surrounding physical and social environment, suggesting that the sense of touch serves as a sensory anchor on which the bodily self extends (Bremner and Spence, 2017) and a channel of communication with other individuals (Morrison et al., 2010). Indeed, the sense of touch is more than a sensory input for haptic exploration, identification, and manipulation of objects in the environment (sensory-discriminative dimension), it also represents one of the most direct means of contact and social interactions, allowing positive and rewarding experience of tactile sensation (affective-motivational dimension; McGlone et al., 2007). More specifically, each tactile experience is processed in terms of sensory-discriminative properties that specify precise information about the spatio-temporal dynamics of mechanical stimulation and texture features of the external object touching the skin toward activation of fast-conducting myelinated Aβ afferents that project to somatosensory cortical areas, and in terms of socio-affective properties that specify the internal state of the organism (e.g., how the experience of been touched feels like; Bremner et al., 2012; McGlone et al., 2014). This second dimension of touch, named affective touch, is mediated by a specialized system of slow-conducting, unmyelinated peripheral afferents (C-tactile afferents) that selectively respond to gentle and caress-like touch (Olausson et al., 2008; Löken et al., 2009). More specifically, C-tactile afferents are activated by dynamic tactile stimuli delivered at slow velocity (1–10 cm/s), low force (0.3–2.5 mN) and neutral (skin-like) temperature (Vallbo et al., 1999; Ackerley et al., 2014). Importantly, activation of C-tactile afferents positively correlates with subjective reports of pleasantness (Löken et al., 2009) and elicit implicit positive reactions (Pawling et al., 2017), implying that the C-tactile system is related to positive affect and to the rewarding value of social interactions, thus providing a link between external sensory information and internal affective states in order to support social connection (Morrison et al., 2010). Additionally, C-tactile afferents project directly to the posterior insula and to other crucial nodes of the social−brain network involved in interoceptive and social processing, including the posterior superior temporal sulcus, medial prefrontal cortex, and dorsal anterior cingulate cortex, (Gordon et al., 2013; Voos et al., 2013; Björnsdotter et al., 2014; Morrison, 2016), suggesting affective touch represents a fundamental link between the self and the others. The neurophysiological properties of C-tactile system may thus constitute a privileged peripheral pathway for tactile stimulation that is likely to act as a selector for picking out and encode socially relevant touch, reflecting a disposition of seeking close affective contact with others and to maintain social bounds (Morrison et al., 2010). Notably, interpersonal affective touch has been shown to critically contribute to communicative behavior and social cognition processing (Olausson et al., 2010). Humans are indeed accurate in discriminating different categories of emotion, even when they are communicated exclusively through touch (Hertenstein et al., 2006). Moreover, interpersonal affective touch can increase the salience of emotional information from other sensory modalities, intensifying the evaluations of other social signals on the basis of the emotional valence of the context. This indicates that the value of touch is intrinsically related to both the physical characteristics of tactile stimulation (i.e., softness, temperature, force, and velocity) and top-down mechanisms that modulate the relevance and affective valence of the stimulation (Ellingsen et al., 2016). Specifically, affective touch has been demonstrated to modulate social appraisal of facial expressions making smiling faces seem more friendly and attractive, and angry faces less friendly and attractive (Ellingsen et al., 2014), suggesting that affective touch can mediate and shape social perceptions in various ways.

### Missing the Touch With the Others: The Growing Problem of Loneliness

The affective and rewarding value of touch in social interactions, mediated by the activation of the C-tactile system, promotes physical contact as a biologically necessity form of stimulation (McGlone et al., 2014). Therefore, one may ask what the consequences of diminished opportunities for tactile social interactions could be. In conditions of physical isolation, people may start feeling a sense of disconnection and loneliness, which critically impact their psychological wellbeing and quality of life. Interesting evidence derives from studies of isolation and confinement, such as Polar expeditions and spaceflights. Indeed, prolonged experiences of physical and social deprivation have been shown to produce psychological changes (e.g., disturbed sleep, negative affect, and interpersonal tension; Palinkas and Suedfeld, 2008) and to impact cognitive ability and brain plasticity. More specifically, reductions in the hippocampal volume of the dentate gyrus were observed from before to after polar expeditions, suggesting that variations in physical and social environments influence hippocampal plasticity, which was associated with lower cognitive performance in selective attention and spatial processing (Stahn et al., 2019). Moreover, decreases in gray matter volume have been evidenced in the right dorsolateral prefrontal cortex and left orbitofrontal cortex, which are involved in executive control, emotional and behavioral regulation (Stahn et al., 2019). Finally, alterations of white matter of the right temporoparietal junction, which is critically associated with social processing, have been found after prolonged isolation (Brem et al., 2020). These results raise interesting questions about the effects of sensory and social deprivation on the brain during periods of isolation. However, it is important to notice that the feeling of loneliness is a more complex experience that goes beyond physical distance. Even though loneliness has been strongly associated with objective physical isolation, this is not a sufficient condition for loneliness, which consists in the subjective feeling of being alone and socially isolated (Russell, 2014). This perceived distance between the self and the others is not necessarily coherent with objective measures of individuals’ social networks (features of the social environment, such as presence/absence of a spouse, amount of contacts with friends and family, participation in social groups), suggesting that some individuals may perceive themselves to be alone even when among other people; on the contrary, others individuals may choose to be alone at times while still feeling connected to others, which is referred as solitude (Hawkley and Cacioppo, 2010). In this view, loneliness is driven by the perceived quality of social relationships and the difficulties in feeling close and connected with social partners, which may be reflected at the neural level by the overlap between self and others’ representations (Courtney and Meyer, 2020). Interestingly, feeling disconnected from others can compromise mental and physical health in both neurotypical and clinical groups (Cacioppo et al., 2006; Kwan et al., 2020) and predict the increased mortality even after adjusting for objective social isolation (Cacioppo et al., 2015). In particular, loneliness has been consistently associated with increased inflammation and higher levels of activation of the hypothalamic-pituitary-adrenocortical (HPA) axis, as reflected by salivary cortisol levels (Cacioppo et al., 2000; Pressman et al., 2005) suggesting that perceived social isolation represents an important stressor for humans. Beside the impact of loneliness on physical health and mortality, it has been shown that perceived isolation dramatically affects also mental health and cognitive functioning (Hawkley and Cacioppo, 2010). Growing evidence indicates that loneliness increases attention to negative stimuli, impacting on emotions, behaviors, and interpersonal interactions, possibly linking loneliness and morbidity through changes in brain structure and function (Cacioppo and Hawkley, 2009). In typically developing preadolescents, feelings of loneliness have been shown to mediate the effect of sociocognitive understanding on depressive symptoms, particularly among girls (Caputi et al., 2017), pointing out the importance of identifying early signs of perceived social isolation in order to prevent an escalation of social deprivation and depressive symptoms. According to a social neuroscience perspective, the behavioral and neural effects of loneliness are related to a short-term self-preservation mode that individuals put to use when they perceive themselves as isolated from the others and therefore, they cannot benefit from the mutual protection and assistance of sociality. That is, loneliness is typically a transient aversive signal that motivates people to become sensitive to potential social threats and renew meaningful social connections needed to survive. However, when social connections are persistently perceived as unavailable, loneliness may become chronic producing deleterious effects on cognition and behavior (Cacioppo et al., 2015). This process results in an increased implicit vigilance for social threats, anxiety, and activation of the HPA axis which carry long-term costs for psychophysiological wellbeing. To mitigate the adverse health effects associated with loneliness, innovative interventions are critically needed to specifically address the risk of clinical manifestations associated with perceived social isolation and sensory deprivation.

Until now, the strategies of intervention have been focused on addressing social isolation by increasing opportunities of social interaction and enhancing social support, and on increasing the quality of social interaction by improving social skills and addressing maladaptive social cognition (Masi et al., 2011). However, to our knowledge, no intervention strategies have been considered sensory aspects. Notably, physical contact and affective tactile exchanges may increase the feeling of closeness and social connection (Morrison, 2016), thus promoting the subjective experience of security and comfort that in tune may result in a decrease of stress reactivity (Ditzen and Heinrichs, 2014). Recent evidence suggests that affective touch reduces feelings of social exclusion indicating that physical contact with others lead to interpersonal connection and social support with stress-protective effects (von Mohr et al., 2017). Moreover, participants exposed to physical contact reported significantly lower neglect scores from their close relationships in a short loneliness scale and they also showed a faster reduction in heart rate, interpreted as a sign of physiological wellbeing (Heatley Tejada et al., 2020). In this respect, developmental studies offer an important understanding on the crucial role of interpersonal affective touch in modulating stress reactivity, creating social bonds and shaping the development of socio-emotional and communicative skills (Cascio et al., 2019). Since the very first stages of life, affective touch is a core self-regulatory and social component of early parent-infant interactions, with the potential of regulate infants’ emotional and physiological state (Stack and Muir, 1992; Feldman et al., 2010; Della Longa et al., 2021b), reinforce social behaviors (e.g., smiling and mutual gaze; Peláez-Nogueras et al., 1997) and facilitate learning of facial information (Della Longa et al., 2019, 2021a), suggesting that early tactile experiences represent the scaffolding of the sense of bodily self and of social connections with others, through which the social brain develops (Montirosso and McGlone, 2020; Farroni et al., 2022). Therefore, it is possible to speculate that including affective touch into intervention programs could have a soothing function particularly in the context of perceived social isolation, buffering the negative effects of loneliness. Particular attention should be paid to developmental age with the aim of identifying early signs of social disconnection and work multisensory interventions up, in order to prevent the potentially adverse effects of stress and social isolation on the brain and to promote physical contact as a crucial neurophysiological substrate that underlies the positive effects of social experiences.

## Interactive Technologies: Do They Connect or Disconnect?

In the last decades, we have started using diverse technological means to communicate and connect with other people, shifting social interactions from in person to virtual-mediated social exchanges. This communication swing has meaningful consequences on different aspects of human interaction, modulating the way people form impressions of one another and come close together (Lieberman and Schroeder, 2020). The massive use of digital technology has attempted to overcome the limits of physical distance providing increasingly more sophisticated devices to connect people who are physically apart, thus making it easier to expand and maintain worldwide social networks. On the other side of the coin, the increased use of mediated communication reduced non-verbal social cues, in particular the opportunities of direct physical contact between people, which may represent a cost in terms of people’s understanding of others’ thoughts and feelings and perceived closeness (Lieberman and Schroeder, 2020). In this view, the fundamental construct of social presence emerges as the sensation that other people are co-present and socially engaged with us within a technology mediated environment with the potential to establish an actual relationship (Biocca and Harms, 2002; Triberti et al., 2018). In a virtual environment, social interaction occurs between representations of others made accessible to the senses *via* technological devices. The salience of the other person in mediated interactions is influenced by intimacy and immediacy of the medium (Short et al., 1976), indicating the importance of the richness of social cues and feedback that the technological devices allowed to be exchanged (Daft and Lengel, 1986). Beside the features of the tools used to virtually communicate, the quality of the relationship should be also considered, focusing on the communication processes that occur in virtual environments as a strategic form of interaction during which people can negotiate and adjust to one another their identities, information and aims (Donath, 1999). The experience of feeling the co-presence of someone else is based on numerous factors including, sensory aspects as well as mutual understanding and behavioral engagement (i.e., inter-agency; Biocca et al., 2003). Therefore, virtual reality is not considered just a communication channel inferior to other forms of interaction anymore, but rather it has been recently reconceptualized as a social space that has its own rules and a great potential to create a meaningful shared world that contributes to give shape to interpersonal interactions and co-constructed relationships (Galimberti et al., 2010). In light of this, the use of interactive technologies may provide a new lens through which to evaluate the effects of different sensory channels and information exchanges on social interaction. More specifically, it is worth considering the role of tactile sensations, asking whether touch can be efficiently included into virtual environments to modulate the sense of social presence and interpersonal connection. This section will critically discuss such open debate. Considering the massive differences between the variety of technologies that make distinct forms of social interaction possible, we will focus on immersive virtual reality (IVR), that is usually delivered through head mounted displays (HMDs), thus blocking out the external world and fully engaging the user in a lifelike experience of free movement, object manipulation, and social interaction (Bohil et al., 2011; Parsons et al., 2017).

### Social Interactions in Virtual Reality

One of the main features that distinguish IVR from other interactive technologies is the possibility of an embodied experience, which increases the sense of presence (namely, the sense of “being there” and being able to enact one’s own intentions) into the virtual environment (Slater et al., 2009; Riva et al., 2014). Embodiment entails the sense of self-location, the sense of agency, and the sense of body ownership toward a virtual body (Kilteni et al., 2012), and is achieved through both realism and fidelity (Zopf et al., 2018). Realism comes from the resemblance to the real body, and depends on whether it takes a first- or third-person perspective, tracks and shadows the whole body or restricted proportions (i.e., hands only, hands, and trunk). Fidelity builds upon the co-occurrence of multimodal stimuli in the same spatio-temporal window (multisensory contingency; Murray et al., 2016), and the correspondence between sensory feedback and motor output (sensorimotor contingency; Baldassarre et al., 2018). The sense of embodiment toward a virtual body has been demonstrated to intensify the emotional processing of the virtual stimuli (Gall et al., 2021). This is particularly relevant for children, who are sensitive to embodiment in a virtual body (Dewe et al., 2021) and tend to truly believe in virtual experiences, sometimes confusing them with reality (Segovia and Bailenson, 2009). Notably, the illusion of body ownership toward one’s own avatar might be enhanced when users receive synchronous visual and affective tactile stimulation, compared to non-affective touch conditions (de Jong et al., 2017). However, the role of affective touch in promoting embodiment remains controversial and would need further investigation (Carey et al., 2021).

Virtual bodies are also employed to create a sense of co-presence with an interactive partner, namely the feeling of being there with a “real” person (Oh et al., 2018). The virtual partner can be either an avatar, namely a virtual representation of a real human user who interacts online, or a virtual agent, which is a digital animation that behaves in a pre-specified way or is controlled by the computer (von der Pütten et al., 2010). People are able to discriminate between virtual avatars and agents, with avatars being more easily identified as such and perceived as likeable, thus inducing higher levels of co-presence and emotional activation (Hoppe et al., 2020). However, also when interacting with virtual agents, users’ experience is sensitive to non-verbal communication cues, such as interpersonal distance, which is the comfort space between social partners and depends on cultural norms and individual differences (i.e., level of social anxiety; Kroczek et al., 2020). Fostering parasocial relationships with virtual characters can be a powerful educational tool for children, who are exceptionally open to making these kinds of connections (Brunick et al., 2016).

The modality that enhances the possibilities for social interaction in IVR are the so-called collaborative virtual environments (CVEs), which enable several users to interact with the environment at the same time, being represented by their unique avatar, acting, moving, and navigating the environment independently, thus communicating directly when they are close enough to another user’s avatar. Such communication is mainly verbal and occurs through the audio system of each device but can also involve vision to different extents (facial expressions and body gestures can be implemented depending on how sophisticated the system is). It is therefore possible to use IVR for remote peer interaction (i.e., peers are actively working together on a shared task or activity, but are physically separated), or even in person interaction (i.e., multiple users work on the same virtual activity, but also share the real space). It is a fascinating option to promote learning from childhood (Bailey and Bailenson, 2017), and also for children with atypical development (Parsons and Cobb, 2011).

### Impact of Interpersonal Virtual Interactions on Loneliness

Like any innovation, IVR has opened the debate about possible social consequences, eliciting on one hand fear and resistance to change and on the other enthusiasm and great expectations. Since Internet and digital technology has become a pervasive means of communication, researchers have begun to investigate the social impact of new interactive technologies on people’s network of relationships and related levels of loneliness (Coget et al., 2002). On one hand, IVR may represent the ultimate connecting tool, which increases the realness of virtual communication providing people with new opportunities to meet and communicate with people physically distant. On the other side, critics of the IVR point out its possible opposite effect of disconnecting people from their bodies and reducing face-to-face interactions. In this way, IVR seems to have the potential for both positive and negative effects on psychological wellbeing and social connection, as people may experience a paradoxical situation in which mental and virtual mobility counterposes physical distance and social separation (Daniel et al., 2018), resulting in a tension that is gradually changing social communication and interpersonal interactions (Lieberman and Schroeder, 2020). Specifically, there is not yet a consensus about the impact of virtual communication on loneliness and psychological wellbeing (Orben and Przybylski, 2019; Odgers and Jensen, 2020). Higher feelings of loneliness among young people have been shown to predict an increased social media use to keep in touch with peers and family, however, it was not associated with happiness (Cauberghe et al., 2021). Some studies found that young adults who use more social media seem to feel more socially isolated than their counterparts with lower social media use (Primack et al., 2017), while other studies suggest that high social media attitudes were associated with decrease of reported loneliness among college students (Pittman, 2015) as well as older adults (Shillair et al., 2015). The inconsistency of results suggest that other variables may mediate the effects of interactive technologies on loneliness, such as the perceived intimacy that makes people feel connected when interacting through virtual technology (Pittman, 2018). Indeed, people’s belief that virtual platforms are a good way to connect with others has been shown to be a more meaningful predictor of decreased loneliness than the frequency of social media use (Pittman, 2018), suggesting that the emotional benefits of virtual communication critically depend on perceiving a real connection and intimacy with the other. Therefore, besides the great opportunity to create an extensive social network, it is possible that people interacting through IVR might still feel alone and perhaps find it difficult to create meaningful social interactions. Moreover, it is also worthy considering possible negative interactions that may take place in virtual environments, including experiences of harassment, bullying and minors’s exposure to inappropriate content (Maloney et al., 2020). Indeed, a participatory observation study revealed similar aspects of bullying found in social VR compared to traditional games, which may be particularly risky given the online anonymity (Maloney et al., 2020). In this regard, high levels of engagement may represent a risk especially for the developmental population and should be carefully examined to design virtual spaces that can guarantee safer and more socially satisfying virtual experiences (Maloney et al., 2020). Additionally, virtual exchanges can induce experiences of cyberostracism, which is an act of social ignoring and exclusion occurring in a virtual environment that lead to negative feelings, reduced perception of control and losing a sense of belonging (Williams et al., 2000). In this context, tactile stimulation has recently drawn attention as a valuable means of social bonding that can modulate the perception of social separation or rejection. Indeed, it has been shown that affective touch is effective in reducing negative feelings of social exclusion experienced during a computer ball-tossing game specifically manipulated to induce ostracism, pointing to a soothing function of touch in a situation of virtual social rejection (von Mohr et al., 2017). In this perspective, tactile interchanges may represent a critical aspect to ameliorate the sense of social presence and intimacy between people interacting in a virtual environment to the aim of creating a positive and enjoyable virtual social experience. The new challenge of developing multisensory and more immersive social VR platforms raises interesting questions about the impact of virtual tactile interactions on the perceived virtual version of oneself identity and the capacity to create self-other meaningful connections. The next paragraph will explore possible benefits and challenges in including tactile stimulation in IVR.

### Bringing Interpersonal Affective Touch Into Virtual Reality

Being primarily visual and auditory, the virtual experience is usually impoverished of touch, a communication channel with unique potential for social interaction and connection. Over the last years, researchers tried to bring touch into virtual experience by understanding the many different aspects of tactile processing and communication across the body surface (Gallace et al., 2007). The world of art and dance is one of the most receptive to the potential of virtual touch, which has been employed to engage people in shared IVR, while also being in the same physical space. This interactive modality embeds the possibility for shared experiences between co-present bodies that can touch one another (Thomas and Glowacki, 2018). Despite the promising complementarity between “real” tactile, proprioceptive, interoceptive sensations, and the primarily visual and auditory inputs of IVR, the combination of modalities has received little consideration in the literature (Cerritelli et al., 2021). Therefore, little is known about the potential effects of receiving real human touch while immersed in IVR. This is probably since this line of research has taken as its main challenge that of fixing IVR as a remote communication tool, which is therefore independent of the physical proximity of the users. To this end, many attempts are being made to design and develop hardware and software that can encode, reproduce and communicate, or simulate interpersonal affective touch. Importantly, different tactile devices induce different feelings, with force feedback actuators being evaluated as more natural and resulting in greater co-presence and emotional sharing than vibrotactile devices (Ahmed et al., 2016). When touching in IVR, people adjust the touch intensity according to the target (i.e., less force for touching virtual agents than objects, less force for touching the agent’s face than the torso area, less force for touching male than female agents; Bailenson and Yee, 2008). On the other hand, both virtual agents and avatars are perceived as having higher agency in case they can touch the users through an artificial hand, which also make participants reporting increased co-presence (Hoppe et al., 2020). Overall, mediated or computer-generated affective touch can intensify the perceived social presence of remote partners, modulate physiological responses, increase trust and affection, help connecting humans and virtual characters, and foster prosocial behaviors (van Erp and Toet, 2015). Indeed, affective touch is fundamental in giving life to the virtual experience, as it is closely linked to emotions, in a mutual influence that nurtures social encounters. When asked to express emotions through handshaking of haptic devices, participants’ kinematics are able to differentiate distinct emotions, which allows other participants to receive another person’s handshake *via* haptic devices and capture its emotional content (Bailenson et al., 2007). Moreover, researchers asked neurotypical adults to observe virtual agents’ emotional faces while seeing a virtual representation of their own hand touching the virtual partner. They were instructed to touch the agent’s hand by squeezing a controller, and to use the same force as when touching a real person, using the same type of touch regardless of the agent’s emotion. Participants applied more force when the agent expressed negative or aroused emotions, and this was mediated by the participant’s own emotional and physiological state (Ahmed et al., 2020). Therefore, people implicitly use touch to communicate (and potentially share) emotions not only in reality, but also in virtual environments and with virtual agents. Indeed, romantic couples have been asked to engage either in a video call or in a video call enriched by the use of a remote massage device. Participants could either send (through the manipulation of a shoulder-like device) or receive (through vibrotactile stimulation) massages. The inclusion of touch increased the perceived emotional and physical connection within the couples (Haritaipan et al., 2018).

Despite all the potential benefits, the implementation of interpersonal tactile devices in VR also raises some risks and ethical issues that should be carefully considered. Introducing tactile stimulation into IVR, the virtual experience tends to connect very closely to the person’s perceptual system, thus allowing for high degrees of immersion. This increased realism of multisensory virtual exchanges fosters more intense experiences, which might result in some people beginning to have difficulty differentiating from the offline and online world. Consequently, cognitive, emotional and behavioral disturbances may arouse after re-entry into the real world following the VR experience (Behr et al., 2005). As the perception of one’s own body can be manipulated in VR, continued exposure to such embodied experiences enriched by somatosensory stimulation, may cause confusion in people about their real body, impacting the development of oneself identity (Slater et al., 2020). Moreover, social interaction in VR could become more enjoyable and desirable in various ways in comparison to real-life interaction, so that people withdraw from society (Slater et al., 2020). This risk is particularly relevant for children or adolescents, who may not distinguish well between reality and virtual reality as adults do (Maloney et al., 2020; Tychsen and Foeller, 2020), and for vulnerable people, such as those prone to psychosis. Although interpersonal exchanges in VR are based on virtual sense data and virtual actions, they are nevertheless real as first-person experiences, thus they have physical, emotional, and cognitive consequences, which may be beneficial or harmful (Slater et al., 2020). Therefore, careful attention needs to be paid to the introduction of interpersonal touch in virtual environments. As well as emphasizing the positive aspects of increasing social connection, potential negative effects should also be considered, specifically in contexts where touch could be unwanted, unpleasant, improper or felt as a violation. Indeed, researchers must take into account specific low-level properties of tactile devices (e.g., how users can control pressure, speed, temperature, and attrition of tactile interactions) as well as the high-level aspects such as communicative intentions and subjective perception (e.g., touch provided by an avatar or virtual agent, context of social interaction, and pleasant/unpleasant feelings). Different effects of virtual tactile interactions should be experimentally evaluated using suitable neurophysiological and behavioral measures to compare participant socio-emotional and behavioral outcomes before and after a brief period in VR, perhaps comparing two scenarios that elicit contrasting emotions. Scientific support will be essential for developers of VR environments and legal authorities to determine evidence-based regulations and recommendations in order to prevent danger or immoderate use of VR (e.g., clear warnings and minimum age requirements). In addition, developers and users need to be aware about the ethical implications and the potential advantages and dangers that can arise as a consequence of interpersonal exchanges in multisensory VR. In particular, to reduce the risk of harm, developers could build up different multisensory levels of virtual interaction that could be flexibly selected and users’ education should also include training of disengagement from VR (e.g., remind users of their right and ability to shut-off the devices and stop the experience at any moment).

## Using Virtual Realities on Social Purpose: How to Foster Social Connection Among the Most Disconnected Individuals

**FIGURE 2:**
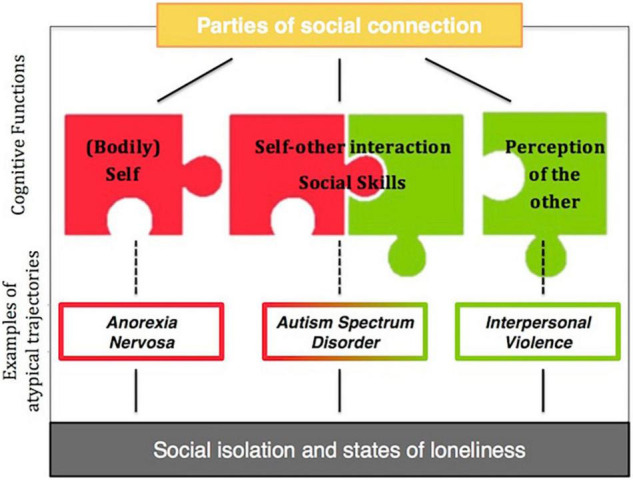
Example cases of atypical connection with the self and the others which may be related to social isolation and states of loneliness. Anorexia Nervosa as a case of disconnection from the bodily self, Autism Spectrum Disorder as atypical self-other interaction and Interpersonal Violence as a result of disconnection from the other.

A number of clinical conditions entails concurrent distorted connection with the self and the others. Early sensory mechanisms and higher-order cognitive and social difficulties are bound together in shaping how people perceive their own bodies and interact with others (Baum et al., 2015). In this manuscript, we review the extant literature and propose that an altered perception and use of touch during self exploration and social exchanges might be part of the neurocognitive and sensory loneliness that affects the social life of some individuals. Research in this area dramatically neglected to investigate the sensory factors and effects of loneliness across different developmental trajectories, clinical conditions, or other cases of social disconnection. In this section, we discuss these aspects in three example cases: anorexia nervosa, autism, and interpersonal violence. Specifically, we select these examples to disentangle three parties of analysis that characterize the ability to create meaningful social connections: the (bodily) self, self-other interaction, perception of the other (Figure 2). Beyond the profound differences among these conditions, a comparative approach allows us to explore how they all include sensory atypia and states of loneliness that impact connection with the self and the others. As the neuroconstructivist approach suggests, early, domain-general, low-level processing deficits might affect several domains but in varying ways and times across different developmental trajectories. Therefore, similar behavioral outcomes may stem from very different cognitive and neural causes, and similar atypicalities may give rise to different manifestations (Karmiloff-Smith, 2009). This gives us some insights into the possibility that sensory mechanisms (i.e., affective touch) across various diagnoses (i.e., Autism Spectrum Disorders and Anorexia Nervosa) and social dynamics (i.e., interpersonal violence) may play a key role in understanding the individual and defining personalized intervention. More specifically, identifying early predictors of loneliness by means of understanding their low-level origins is of great importance to design sensory interventions through careful identification of which level of stimulation is appropriate to promote social connection in different individuals through each sensory modality. This approach would open new perspectives for individuals at heightened risk for or with impairments in social abilities, as well as those experiencing high levels of loneliness, both during development and in adulthood. In this perspective, technologies have unique advantages and potential to manipulate through the senses, the perception that we have of ourselves and others. In the next paragraphs, we will present some applications of these theoretical approaches.

### Disconnection From the Bodily Self: Anorexia Nervosa

Anorexia Nervosa (AN) can be considered a clinical example of disconnection from the bodily self which comes with a vicious cycle of social isolation, loneliness, and disconnection from others (Levine, 2012). Patients suffering from this condition not only present low body weight and behaviors to avoid gaining weight (American Psychiatric Association, 2013), but also present a maladaptive need for control, scarce flexibility, low emotional awareness and expression, reduced connection, and intimacy with others (Hempel et al., 2018). Notably, most patients with AN reported that both core symptoms (i.e., eating and weight concerns, drive for physical activity), loneliness and mood conditions increased during COVID-19 pandemic (Schlegl et al., 2020). As a sensory aspect of social disconnection, AN patients frequently report reduced pleasantness of interpersonal affective touch (Crucianelli et al., 2016). These subjective ratings are sustained by reduced response to affective touch in the brain’s reward and body image systems (Davidovic et al., 2018). Moreover, the atypical processing of affective touch persists after patients recover from AN (Bischoff-Grethe et al., 2018; Crucianelli et al., 2021), indicating that sensory mechanisms have long lasting effects on people’s sense of self and connection with others. On the other hand, preliminary findings support the use of massage and social touch for people with AN, showing that the inclusion of massage therapy in a standard treatment reduces the stress and anxiety levels and decreases the scores of body dissatisfaction (Hart et al., 2001).

Innovative interventions to modulate the individuals’ sense of bodily self, which happens to be atypical in AN, are based on virtual body illusions that manipulate multisensory body-related signals. Specifically, synchronous visuo-tactile stroking of the real and virtual body has been used to achieve full body illusions and make people feel embodied in a virtual body that may differ in shape and size from the one’s own real body (Petkova and Ehrsson, 2008). Through the illusory sense of ownership over the virtual body, the mind also generates attitudes and behaviors that are congruent with that type of body (Slater and Sanchez-Vives, 2014). Interestingly, it has been found that people with AN are more susceptible to bodily illusions compared to healthy controls, with enhanced sensitivity for visual manipulations of the body self (Crucianelli and Filippetti, 2020). This led researchers to investigate the beneficial use of IVR to treat eating disorders through the full body illusion (Riva et al., 2021). The visuo-tactile stimulation of the real and virtual body is an effective way to make individuals with AN feel as they own the virtual body, thus allowing the assessment and modification of their body image (Serino et al., 2019). Such exposure and embodiment in a virtual body can decrease the overestimation of AN patients’ own body size (Keizer et al., 2016). Given the strong connection between the bodily self and social abilities, IVR might be used not only to directly re-shape the patients’ body image, but also to leverage such rehabilitative embodiment to engage people in “rehabilitative social interactions.” Indeed, recent therapeutic approaches specifically target AN interventions to social connection (Hempel et al., 2018). To this end, integrating interpersonal affective touch in IVR applications for the treatment of AN would provide a sensory framework to simultaneously manipulate the bodily self and connect the bodily self with the others throughout physical contact. Immersive technologies would have the unique possibility to adapt tactile stimulations to the individual’s sensory perceptions and feelings of pleasantness, thus allowing patients to receive and deliver affective touch in a safe and controlled environment, discover what type of interpersonal touch they like or dislike, and potentially expand the range of affective tactile sensations perceived as positive. We propose that with this bodily and social experience one can also take care of core symptoms of distorted body image.

### Self-Other Disconnection: The Case of Autism

From early childhood, children with NeuroDevelopmental Disorders (NDDs) show early risk markers of atypical sensory processes, which confer cascading effects on child development, potentially marking the onset of neurodevelopmental difficulties and disorders (Hill et al., 2012) and being the very first source of social disconnection. Children with NDDs are the most exposed to social exclusion, interpersonal disconnection, and loneliness, which is a predictor of mental health issues later in life (Kwan et al., 2020). They frequently manifest hypo or hyper-responsiveness to tactile stimulation and avoidance or seeking behaviors toward touch (Smirni et al., 2019), which has the unique power of connecting the self with the others in an indissoluble bond between touching and being touched. In particular, Autism Spectrum Disorders (ASD) are persistent and pervasive deficits in social communication and social interaction, as well as restricted and repetitive patterns of behaviors, interests, or activities (American Psychiatric Association, 2013). In adulthood, loneliness seems to mediate the effects of autism features and social contact on mental health (Schiltz et al., 2021). Looking at the sensory aspect of self-other disconnection, individuals with ASD have unique (and heterogeneous) processing of tactile stimuli (Balasco et al., 2020), such as atypical brain responses to both affective and non-affective touch (Kaiser et al., 2016), delayed or reduced effects of visuo-tactile stimulation on the bodily self (Cascio et al., 2012; Greenfield et al., 2015), reduced subjective pleasantness of affective touch (Voos et al., 2013). Notably, touch deprivation is associated with altered sensory thresholds, depression, and self-aggression (Field, 2005), which are frequent symptoms of ASD. On the other hand, people with ASD seem to benefit from intervention through massage and social touch (Rodrigues et al., 2019). A recent study suggested that 5 months of daily parent-delivered massage and weekly sessions of therapist-delivered massage brought great benefits to preschool children with ASD. When compared to no-treatment controls, children showed more pronounced improvements in sensory responses, self-regulatory abilities and receptive language, as well as reduced symptom severity (Silva et al., 2015). A longitudinal follow-up study confirmed the massage beneficial effects over the long term (Silva and Schalock, 2016).

Technologies such as immersive virtual reality become increasingly popular to reshape sensory and bodily experiences and social connection for clinical goals, with long-lasting effects that generalize to the real world (Riva, 2022). For instance, IVR is largely employed to deliver social skills training in safe, various and ecological situations (i.e., classroom, park, shop, and street), where individuals with ASD can foster their communication (verbal and non-verbal), social cognition (i.e., Theory of Mind – ToM), emotional competences (i.e., emotion recognition and regulation and empathy), as well as learn appropriate social behaviors (Herrero and Lorenzo, 2020). However, results about IVR social training are still preliminary in providing evidence of effectiveness, duration of effects, transfer of skills to real-world contexts. Moreover, the virtual experience is not a perfect replica of the one we have in reality but is rather different in both bottom-up sensory aspects and top-down cognitive mechanisms (Harris et al., 2019; Giesel et al., 2020). This becomes particularly relevant when we talk about people with atypical development and unique sensory functioning. For instance, real and virtual experiences might have distinct sensory implications for people with ASD (Valori et al., 2021). The implicated sensory mechanisms might also affect cognitive and social processes, as suggested by the evidence that people differently adapt their social behaviors (i.e., interpersonal distance) in real or similar IVR environments, but this does not seem the case for people with ASD, who show similar behaviors in the two environments, toward a real partner or virtual avatar (Simões et al., 2020). In addition, it has been suggested that adults with ASD are less susceptible to the full body illusion in IVR, which was associated with more severe autistic traits and social difficulties (Mul et al., 2019). This may indicate a reduced sensitivity to visual manipulations of body self, which would limit the possibility of intervention through the visual channel. On the other hand, people with ASD seem to heavily rely on somatosensory cues (Izawa et al., 2012), of which touch is particularly powerful in connecting the self and the other. The potential of leveraging tactile inputs in IVR to foster the body illusion, shape the bodily self and stimulate social connectedness of people with ASD has yet to be investigated.

In an attempt to integrate touch in applications for ASD, literature describes many prototypes of virtual tactile tools designed for telemedicine (distant therapy) or innovative intervention. The idea here is to develop tools that can deliver tactile stimuli with no role of human partners, or with remotely interacting partners. Vaucelle et al. (2009) designed tools such as Touch Me and Squeeze Me. With the former, caregivers can remotely activate a vibrotactile motor array to deliver tactile inputs to large areas of the patient body. With the latter, both caregivers and patients themselves can use a digital control system to hug the patient who wears a sort of tactile vest. The authors mention that these technologies are also suitable for people with touch aversion, thus allowing them to experience touch without the overwhelming human contact (Vaucelle et al., 2009). Overall, researchers created tactile technologies to help people with ASD experiencing human contact through virtual simulations of being touched (Tang et al., 2014). To the best of our knowledge, these studies rarely go beyond specifying design features, creating prototypes, assessing feasibility, and piloting. A deep understanding of the subjective, behavioral, physiological, and neural responses of people with ASD (also taking their profound interindividual differences into consideration) of such simulated touch has yet to be conquered. Beyond the main barriers imposed by the limitations of technology in integrating touch into IVR, it is important to note that also theoretical hurdles arose from the idea that people with ASD rely on a primarily visual learning style, and this would blend happily with the primarily visual (and auditory) characteristics of virtual worlds (Strickland, 1997). This prevented researchers from wondering whether VR has any unique potential for stimulating touch in individuals with ASD. Future research could explore innovative ways to adapt tactile stimulations to the individual’s functioning and needs. In this respect, IVR offers unique options to manipulate the stimulation to re-shape sensory thresholds, bodily perceptions and feelings. Simultaneously, these low-level manipulations could lay the foundations to allow people with ASD to prove themselves in interpersonal interactions that are tailored to the personal needs of each individual, who can be facilitated in his/her discovery of pleasant affective tactile experiences.

### Disconnected From the Other: Interpersonal Violence

One of the worst-case scenarios for failure to connect with others is interpersonal violence, which is the violence inflicted by one individual to another (Dahlberg and Krug, 2006). This social dynamic involves two parts, the victim, and the perpetrator of violence, who both may experience forms of loneliness and difficulties in connecting with others. Looking at the victims of interpersonal violence (i.e., bullying), they report higher levels of loneliness compared to controls and offenders, from early in childhood and across cultures (Eslea et al., 2004). The violence suffered frequently results in post-traumatic stress disorders (PTSD) that lead the victims to ambivalent perceptions, feelings and thoughts toward affective exchanges (Eslea et al., 2004). Such trauma profoundly affects the psychological functioning of the victims, beginning with their sensory responses to social stimuli. For instance, victims of interpersonal violence and PTSD show aversion and atypical neural activation for skin-to-skin touch (Strauss et al., 2019). On the perpetrator’s side, it has long been suggested that violent behaviors could be modulated by feelings of loneliness (Check et al., 1985). Notably, loneliness affects the perception of others as distant or disconnected and may contribute to the tendency to see them as less fully human than the self (Haslam, 2022). In line with the strong interconnection between social and perceptual mechanisms, recent studies suggested that aggressive behaviors are associated with reduced ability to discriminate emotions in faces (Zeng et al., 2021), with male offenders having difficulties toward female fearful faces (Nyline et al., 2018), and a bias toward classifying fear as happiness (Seinfeld et al., 2018). On a positive note, this dehumanization process might be reduced by promoting social connection, which has been done through sensory stimulation such as embodiment in virtual scenarios and interpersonal affective touch.

The possibility to immerse the senses of one person into the eyes of the other person is a powerful way to fight interpersonal disconnection and the resulting manifestations of violence. To this aim, IVR has been recently used to investigate and train individuals’ ability to connect with others from the outgroup. Researchers found that when observing interpersonal violence between virtual humans interacting in IVR, participants intervened physically to help the victim more frequently if the victim was from the same social group. They were also more sensitive to the ingroup victim’s gaze for help (Slater et al., 2013). As a perceptual perspective-taking training, IVR has been used to induce a full body illusion that brings men in the female body of a victim of domestic violence. After being embodied in a female victim and exposed to violent scenarios, offenders improved their ability to recognize fearful female faces (Seinfeld et al., 2018).

Scientists have been suggesting for some time that the lack of affective touch in child nurture is leading to an increase in violence against ourselves and others across the lifespan, while placing a high emphasis on touch in childhood results in a lower incidence of violence among adults (Field, 2005). This evidence points to interpersonal affective touch as a promising complement of traditional interventions. Indeed, touch has the potential to shape our perception, emotions, cognition and attitudes toward others, thus reducing self-other boundaries in interpersonal and intergroup interactions (Shamloo et al., 2020). This is particularly powerful when people interact with social partners who are perceived as different from them on salient aspects (i.e., ethnicity, gender and so on), that might be a risk factor for interpersonal violence. A possible application consists in massage therapy, which may promote well-being and reduce aggressive behaviors, through reducing cortisol and increasing serotonin levels (Field, 2005).

In light of these considerations, we can speculate on the potential of including affective touch in IVR interventions for victims and perpetrators of social violence. While interacting with virtual agents (namely computer-controlled characters), the way individuals touch the other seems to mirror their interpersonal attitudes. For instance, by using controllers to hug virtual agents, participants differently modulate touch duration and intensity according to their own gender and attitudes toward their own and others’ bodies (Tremblay et al., 2016). The combination of IVR and interpersonal touch may further boost the possibility to foster social connection through embodied self-other experiences. The resulting process of identification, differentiation and comparison between oneself and the others, first takes place in the bodily domain (Meltzoff, 2007; Tsakiris, 2016) and in tune extends to socio-cognitive domain, resulting in reduction of implicit biases against outgroup members and modulation of social cognition processing (Paladino et al., 2010; Farmer et al., 2014). Indeed, during interpersonal interactions, shared sensory experiences may partially modulate the overlap in the brain representation of the self and the other, which underpin the basis of social understanding and social connection (Brozzoli et al., 2013; Courtney and Meyer, 2020). These findings suggest that sensory experiences shape the representation of one’s own body as a point of reference for interactions with the external social environment with cascading effects on socio-emotional and cognitive development. Future studies could deepen the role of interpersonal affective touch to assess the modes of interaction and communication deployed by victims and perpetrators of interpersonal violence. This would provide a deeper understanding of the sensory and relational mechanisms related to violence, and open new perspectives on intervention mediated by physical contact and affective touch.

## Discussion

The present review offers an innovative and multidisciplinary perspective on the human need for social connection in a world that relies more and more on distant communication. Specifically, we focused on the impact of interpersonal affective touch, as an essential means of social connection that may increase the sense of social presence and emotional support during virtual exchanges, thus preventing possible aversive effects of feeling lonely and disconnected from the others. Indeed, with the extensive diffusion of digital technologies, an increasing amount of social interaction is mediated by communication devices, substituting direct physical contact (Twenge et al., 2019), which on one hand facilitates communication between distant people and on the other may reduce opportunities of physical contact critically impacting the ability to establish emotional and meaningful social bonds. Therefore, one of the most challenging future directions for the IVR field is the integration of interpersonal touch in virtual realities, critically supporting the human need to feel emotional and social connection through physical contact. This would expand the perspectives to manipulate users’ perception beyond the possibilities given by visual and audio stimulation. In this perspective, the open debate about the consequences of interactive technologies on social interactions should move from asking whether new technologies connect or disconnect people, to investigating different forms of social connection based on multisensory exchanges through innovative tools.

To date, researchers mainly used IVR and tactile stimulation to achieve body ownership illusions toward the virtual body, thus manipulating individuals’ sense of self or attitudes toward others (i.e., members of out-groups; Maister et al., 2015). However, this interactive technology can go far beyond this, and enable different types of interpersonal interactions and connection. For example, haptic interfaces may enable tactile communication between people who are physically apart by providing mediated interpersonal affective touch, which can carry important socio-emotional feedback. Overall, advances in technology are still a long way from offering effective and accessible proposals for integrating touch into the virtual, especially social, experiences. For instance, haptic devices lack physical cues such as temperature, grip, textures, and limit users’ emotion discrimination as compared with *in vivo* human touch (Bailenson et al., 2007). However, we are not facing a mere technical challenge. Future research should carefully investigate the behavioral, psychophysiological, and neural responses that are elicited by any future devices that would bring interpersonal affective touch in virtual realities. Although it is still under discussion to what extent mediated affective touch can reproduce real interpersonal tactile interactions, there is preliminary evidence supporting that physiological, behavioral and social reactions to mediated touch resemble the way people experience and react to direct interpersonal touch (Bailenson and Yee, 2008). Interestingly, also the representation of the space surrounding the body (i.g. peripersonal space) has been shown to adapt because of technology-mediated and social interactions (Serino et al., 2018; Serino, 2019).

Besides the fascinating perspective of studying how to invent tactile technologies to simulate interpersonal touch, we also have the intriguing potential of using concomitant immersion in IVR and “real” skin-to-skin contact with others, whereby users share the same physical space and discover completely new means of interaction. The different effects of simulated and real touch remain largely unexplored, as well as the role of contextual factors, such as the identity, intentions and emotional profile of the person who is touching us or touched by us (i.e., AI avatar, remote human partner represented by the avatar). This is particularly powerful when people interact with social partners who are perceived as different from them on salient aspects (i.e., ethnicity, gender and so on), that might be a risk factor for scarce social connection. Interpersonal touch represents a multisensory experience that involves bottom-up processes (the neurophysiological properties of affective touch, mediated by the activations of C-tactile afferents), as well as top-down processes (i.e., the other familiarity, our past experiences and expectations) that modulate the emotional valence of physical contact between individuals. The identity, intent and relationship with the person delivering the touch become part of a complex interplay of many sensory, emotional, and social factors, which ultimately determine the perceptive experience and communicative meaning of tactile interactions. However, to the best of our knowledge, the applicability of interpersonal tactile intervention in VR interactions has never been investigated as a potential driver of social connection.

From a clinical perspective, the use of new technologies that provide additional tactile stimulation could be of high potential in healthcare. More specifically, tactile stimulation, which is frequently used by therapists for patients suffering from various conditions that benefit from massage or manipulative treatment of tissues, can also be beneficial for people who suffer from different forms of social disconnection (e.g., patients in physical isolation or quarantine, lonely people, individuals refusing touch by another person). Indeed, recent evidence suggests that physical contact has beneficial effects on reported feelings of loneliness (Heatley Tejada et al., 2020) as well as positive physiological effects (Jakubiak and Feeney, 2017). In addition, IVR offers unique opportunities to assess and manipulate individual profiles of sensory processing, affective tactile interaction, bodily self, and social abilities. This opens new perspectives to intervene on those cases where atypical functioning of these interrelated mechanisms is associated with clinical conditions or interpersonal disconnection. We have presented evidence about autism and anorexia nervosa, which entails an atypical sense of self and social disconnection, as well as interpersonal violence as a worst-case scenario of social disconnection. We critically reviewed the extant literature and proposed speculations on the way affective touch and IVR can be used for people with such conditions. Beyond the massive differences across these example cases, we believe they all involve multisensory atypia, which affect not only the sense of self but also the difficulty in connecting with others, possibly resulting in feelings of loneliness. Our considerations might also apply to other examples of psychopathology and interpersonal dynamics. In this respect, it is worth mentioning that there is not one unique pattern of affective touch processing by individuals, and researchers and clinicians aiming at the design and implementation of IVR training should be aware of the individual processing styles of the target users to effectively tap their needs, strengths, and weaknesses. Specifically, to address the need of interventions aiming to mitigate the negative effects of sensory deprivation and social isolation, innovative initiative should foster cross-disciplinary collaboration, combining advances in technology with psychophysiological assessment in order to rapidly and efficiently translate knowledge, methodologies and technologies from laboratory experiments to clinical applications. The continuation and extension of this approach is a key factor to help bridging the gap between academic researchers investigating psychological aspects and digital technology developers. Future collaborative research initiatives could lead to better understanding of mechanisms underpinning loneliness and social disconnection, providing the basis to develop efficient and innovative assessment tools and personalized treatment interventions to prevent long-term health consequences of perceived social isolation across different clinical conditions.

## Conclusion

In conclusion, interpersonal exchanges in IVR are not a mere simulation of real interactions but can rather offer alternative modes of contact with the bodily self and with the others. In particular, the integration of interpersonal affective touch in virtual interactions has the potential for leveraging an innovative way to connect people and create diverse forms of social participation. This challenging perspective would push our possibilities of social connection into a virtual space, thus reshaping our understanding of multisensory interpersonal interactions and offering new opportunities for advances in interactive technology applications and clinical interventions with both developmental and adult populations.

## Author Contributions

LD provided the conceptualization. LD and IV contributed to discussing and wrote the original draft of the manuscript. LD, IV, and TF reviewed and edited the manuscript and approved the submitted version.

## Conflict of Interest

The authors declare that the research was conducted in the absence of any commercial or financial relationships that could be construed as a potential conflict of interest.

## Publisher’s Note

All claims expressed in this article are solely those of the authors and do not necessarily represent those of their affiliated organizations, or those of the publisher, the editors and the reviewers. Any product that may be evaluated in this article, or claim that may be made by its manufacturer, is not guaranteed or endorsed by the publisher.
