# Supplementary material for: Interpersonal Affective Touch in a Virtual World: Feeling the Social Presence of Others to Overcome Loneliness
Source: Front Psychol. 2022 Jan 11;12:795283. doi: 10.3389/fpsyg.2021.795283 (PMC8787079; doi:10.3389/fpsyg.2021.795283)
Supplement: Supplementary file 1 [file Table_1.PDF]

SUPPLEMENTARY MATERIALS

Table summarizing the main studies discussed throughout the narrative review, showing characteristics and methods and the main outcomes of each study. Studies have been reported according to the order of citation in the manuscript and divided by paragraphs. The topic of the first section (blue) is **"Social connection through tactile experience"** and it includes the paragraphs "Neurophysiology of interpersonal affective touch: a bridge between the self and the others" and "Missing the touch with the others: the growing problem of loneliness". The topic of the second section (yellow) is **"Interactive technologies: do they connect or disconnect?"** and it includes the paragraphs "Social interactions in virtual reality", "Impact of interpersonal virtual interactions on loneliness" and "Bringing interpersonal affective touch into virtual reality". The topic of the last part is **"Using Virtual Realities on social purpose: how to foster social connection among the most disconnected individuals"** and it includes the paragraphs "Disconnection from the bodily self: Anorexia Nervosa", "Self-other disconnection: the case of Autism" and " Disconnected from the other: interpersonal violence"

Paragraph	Keywords	References	Study characteristics and methods	Main outcomes
**Neurophysiology of interpersonal affective touch: a bridge between the self and the others**	Interpersonal affective Touch	Loken et al., 2009	Type of study: microneurography, psychophysical and behavioral.<br>Participants: 25 participants (microneurography study), 20 (psychophysical study), 10 (behavioral study).<br>Method: Microneurography for recording single mechano-afferents (C-T, SAI, SAII, hair, field) during brushing with different velocities (0.1, 0.3, 1, 3 or 10 cm/s) and force (0.2 or 0.4 N). Similar stimulation protocol in psychophysical sessions.<br>Rating the positive hedonic quality of brushing on a visual-analogue scale (VAS).	Results: CT afferents showed an inverted U-shaped relationship between brushing velocity and mean firing rate with highest response at 1,3 and 10 cm/s-1. In contrast, mean firing rate increased monotonically with brushing velocity in all myelinated afferent types. Significant linear correlation between mean firing rates and mean ratings of pleasantness for C-T units, but not for myelinated units (SAI,SAII, hair, field).<br>Limits: hedonic rating also depends on other factors.<br>Conclusions: First demonstration of relationship between positive hedonic sensation and coding at level of peripheral afferent nerve, suggesting that CT fibers critically contribute to pleasant touch. The other afferents showed no relationship with pleasant ratings.
	Interpersonal affective Touch	Olausson et al., 2008	Type of study: stimulation procedure, electrodermal recording.<br>Participants: 2 patients with sensory neuronopathy syndrome, 8 controls.<br>Method: tactile stimuli delivered by the experimenter with a brush at 5cm s-1 speed, on a 10-15 cm distance, with 0.8 N stimulation force). Skin resistance in response to these stimuli was registered with a constant current electrodermal recording device.<br>In addition, participants' ability to identify the brushing location was evaluated.	Results: patients lacking A-beta afferents in the skin area showed CT activation in response to stroking. The stimulation elicited sympathetic skin responses in both subjects, but the mean onset latency of one subject was longer than the ones of healthy subjects. Patients also demonstrated above-chance ability to localize stimuli.<br>Limits: small sample (nearly a case-study).<br>Conclusions: CT-targeted stimuli elicited sudomotor sympathetic response in patients with neuronopathy, despite subjects not reporting sensations of touch in daily life. CT activation may have autonomic consequences despite the absence of perception.<br>The difficulty in spatial localization of stimuli confirms that the CT system is not involved in discriminative touch, whereas the above-chance localization is coherent with the possible somatotopic organization of the insular cortex.

	Interpersonal affective Touch	Ackerley et al., 2014	Type of study: microneurography in vivo, psychophysical measures.<br>Participants: 20 healthy adults (13 females, 7 males) for microneurography + 30 adults (15 females, 15 males) for psychophysiological data.<br>Method: Recording of single CT axons in human participants while being stroked on the skin of the forearm, at five different speeds (0.3, 1, 3, 10 and 30 cm/s-1) and at three different temperatures (18°C, 32C°, 42°).<br>Psychophysical measures: the same procedure was adopted, but here participants rated the pleasantness of the mechano-thermal stimuli with a visual analogue scale (VAS).	Results: CT units were activated vigorously when the experimenter stroked the receptive field with slow, gentle movements and their maximal mean firing frequency occurred at the stroking velocity of 3 cm s−1 and temperature of 32°C. Significant effect of temperature for all stroking velocities (neutral temperature produced significantly higher CT mean firing frequencies).<br>Significant correlation between the CT firing frequency and pleasantness ratings at the neutral temperature only.<br>Limits: not reported.<br>Conclusions: CTs responded vigorously to slow stroking stimuli delivered at typical skin temperature, and CT firing frequencies correlated with hedonic ratings to the same mechano-thermal stimulus only at the neutral temperature.
	Interpersonal affective Touch	Vallbo et al., 1999	Type of study: microneurography.<br>Participants: 38 skin afferences (27 low-threshold units, 11 high-threshold units) collected from 17 participants (8 women and 9 men, age 21:31).<br>Method: lateral antebrachial cutaneous nerve impulse recording with the microneurography. The experimenter employed different tools (glass rods and wooden sticks) to deliver mechanical stimuli, in addition to finger stroking.	Results: based on their sensitivity to mechanical stimuli, units were classified in low-threshold units (27) and high-threshold units (nociceptors; 11). Conduction velocity was estimate from the latency between initial rise of force and the first nerve impulse. The low-threshold group responded strongly to innocuous tactile stimuli, did not discriminate between pin pricks and smooth-probe indentations, showed strong response to slowly moving stimuli but poor sensitivity to fast moving stimuli, intermediate rate of adaptation, a clear fatigue to repeated stimuli and a delayed acceleration during long-lasting stimulation.<br>Limits: not reported.<br>Conclusions: human hairy skin is innervated by highly sensitive mechanoreceptive units with unmyelinated afferents. Their response properties strongly suggest a role in coding some specific features of touch stimuli. Speculative link between the slow tactile system and limbic functions.
	Interpersonal affective Touch	Pawling et al., 2017	Type of study: self-report, electromyography, psychophysiological measures.<br>Participants: 29 young adults (18 women, 11 men).<br>Method: The experimenter strokes the palm or forearm with different touch stimulations conditions. Touch was delivered at speeds of 3 cm/sec * 1 stroke (CT optimal condition), 30cm/sec * 1 stroke (CT non-optimal condition, matched for stroke number) and 30 cm/sec * 10 strokes (CT non-optimal condition, matched for duration). After each trial the participant rated the pleasantness of touch received using a VAS. Participants' Heart Rate (HR)	Results: CT-optimal touch stimulation was rated significantly more pleasant than both type of CT-suboptimal stimulations regardless of where it was applied (forearm vs palm).<br>CT-optimal stroking elicited significantly greater HR deceleration than either type of stroking, regardless of where it was applied.<br>CT-optimal touch induced greater activity in the zygomaticus muscle indicative of a positive affective response, than touch applied at faster velocities.<br>Limits: not reported.<br>Conclusions: stimulation of CT-afferents, on both the palm and the forearm, generates an implicit emotional response, suggesting that

			was recorded during the experimental session.	CT-optimal touch is a more powerfully rewarding stimulus compared to fast touch.
	Interpersonal affective Touch	Björnsdotter et al., 2014	Type of study: fMRI study, touch stimulations.<br>Participants: 22 healthy adults (9 women), 10 healthy children (6 females, range 5.6-13-3 years), 9 healthy adolescents (4 women, range 13.5-17 years).<br>Method: during fMRI scan, tactile stimuli were applied to the forearm (CT-target touch) and to the palm of the hand (Aβ-target touch). participants were instructed to focus on the tactile sensation.	Results: all adult somatosensory regions (left S1, right S2, left S1) activated in response to touch stimulations were significantly activated also in children and adolescents. Adults, adolescents, and children robustly activate the posterior insular cortex in response to CT-touch.<br>Limits: personality traits, social behavior and touch preferences were not controlled.<br>Conclusions: There is continuity, from childhood to adulthood, in the neural processing of sensory-discriminative and affective-motivational touch.
	Interpersonal affective Touch	Gordon et al., 2013	Type of study: fMRI study, touch stimulation.<br>Participants: 22 right-handed adults (9 women).<br>Method: participants received continuous brushing (right palm vs right forearm) and the velocity of the brush was optimal for CT afferents. During the fMRI scan, participants were instructed to close their eyes and to focus on the touch experience.	Results: the perception of CT-target gentle touch is supported by specific mechanisms including the right pSTS, right posterior insula and right mPFC/ dACC. Touching the palm, compared to touching the arm, shows greater activation in the right cerebellum and left parietal cortex. During gentle touch on the arm, compared to touch on the palm, the right mPFC/dACC activation shows greater connectivity with the left insula and amygdala.<br>Limits: not reported.<br>Conclusions: BOLD response to gentle brushing revealed the involvement of a network of brain regions, in addition to the posterior insula, during CT-targeted affective touch to the arm. This network included areas known to be involved in social perception and social cognition.
	Interpersonal affective Touch	Morrison 2016	Type of study: meta-analysis of fMRI studies.<br>Participants: 17 published affective touch studies.<br>Method: PubMed literature search with keyword combinations “affective + touch,” “pleasant + touch,” “touch + emotion,” and “fMRI.”	Results: posterior insula (PI) more likely active for affective touch, and primary somatosensory cortices (SI) more likely active for discriminative touch. Secondary somatosensory cortex associated with both types of touch. Different networks seem related to discriminative or affective touch.<br>Limits:<br>Conclusions: affective and discriminative touch are dissociable both on the regional and network brain levels.
	Interpersonal affective Touch	Hertenstein et al., 2006	Type of study: behavioral, self-reports.<br>Participants: 106 unacquainted dyads.<br>Method: pairs (encoder and decoder) were separated by a curtain. 12 emotions were presented to the encoder, who communicated each emotion by touching the decoder’s	Results: participants showed above-chance accuracy in recognizing some of the emotions (anger, fear, disgust, love, gratitude, and sympathy).<br>Limits: not reported.<br>Conclusions: humans can discriminate different categories of

			bare arm. The decoder had to identify the emotion among 13 response options.	emotion, even when they are communicated exclusively through touch.
	Interpersonal affective Touch	Ellingsen et al., 2014	Type of study: behavioral measure, pupillometry, touch stimulation<br>Participants: 39 participants (20 females, 19 males)<br>Method: 2 sessions, counterbalanced, with 40 IU oxytocin or saline in a double-blind manner. In both sessions participants viewed gray scale images of faces with happy, neutral, or angry emotional expressions. Simultaneously the participants received stimuli (gentle human touch vs vibratory stimulus from a machine). Participants were asked to evaluate faces (friendliness and attractiveness) and tactile stimuli (pleasantness and intensity).	Results: after oxytocin treatment, relative to placebo, human touch sharpened the rating of friendliness and attractiveness of faces. Facial expression of faces shaped pleasantness of human touch strongly than machine touch.<br>Oxytocin did not alter touch experience.<br>Human touch produced larger pupil responses to happy expression of faces than machine touch.<br>Limits: not reported.<br>Conclusions: social information from visual and tactile sources had reciprocal effects on the appraisal of these stimuli.
**Missing the touch with the others: the growing problem of loneliness**	Loneliness	Stahn et al., 2019	Type of study: MRI study.<br>Participants: 9 polar expeditioners (5 men and 4 women) who lived in Antarctica for 14 months + 9 controls.<br>Method: Imaging data and cognitive performance were obtained before and after the mission to study changes in the volume of subsections of the hippocampus and of whole-brain gray matter.	Results: significant reductions in the hippocampal volume of the dentate gyrus from before to after the expedition in the experimental group compared to controls. The reductions in dentate gyrus volume were also associated with lower cognitive performance in tests of spatial processing and selective attention.<br>Limits: small sample size.<br>Conclusions: sensory deprivation and social isolation have deleterious effects on the brain.
	Loneliness	Brem et al., 2020	Type of study: MRI study.<br>Participants: 6 male astronauts + 10 controls.<br>Method: isolation study to mimic a space-mission to Mars. Highly sophisticated selection process.<br>The pre and post 520 days isolation Diffusion Tensor Imaging (DTI) was applied to measure the effects of long-term confinement on white matter microstructure.	Results: reduced fractional anisotropy in participants' scans compared to controls was found in the right hemisphere involving the white matter of temporal, parietal, and occipital lobe as well as the dorsal parts of the internal and external capsule. These differences were most significant in the right temporo-parietal-junction-zone.<br>The longitudinal analyses of 3 participants revealed no significant differences of the DTI-scalars compared to each other.<br>Limits: small sample size, incomplete longitudinal data.<br>Conclusions: sensory deprivation and confinement may account for the regional fractional anisotropy reductions in the right temporo-parietal-junction-zone.
	Loneliness	Courtney & Meyer, 2020	Type of study: fMRI study.<br>Participants: 43 young adults.<br>Method: self- and other-reflection task for 16 targets: the self, 5 close others, 5 acquaintances, and 5 celebrities during a fMRI scan to examined neural responses to the	Results: three clusters emerge from cross-category similarity in mPFC activation: self, social network members (close others and acquaintances), and celebrities. Activation magnitudes in the mPFC linearly increased with the social closeness of the target to the participant. In addition to mPFC, whole-brain searchlight analysis

			self and others in a brain region that has been associated with self-representation (mPFC) and across the whole brain. They reported their subjective closeness to each target and their own trait loneliness (Revised UCLA Loneliness Scale).	revealed a cluster in PCC/precuneus that extended into temporoparietal junction, middle temporal gyrus, and temporal poles.<br>Greater loneliness was associated with less mPFC activation. The neural similarity of social targets decayed with increasing distance between the social categories in mPFC and PCC. Loneliness was associated with blurred boundaries between the social circles surrounding acquaintances and lonelier individuals represent others as more distant or dissimilar from the self in the mPFC<br>Limits: difficulty in precisely testing for overlapping representations between the self and others.<br>Conclusions: participants who were less socially connected showed altered self-other mapping in social brain regions. Loneliness was associated with reduced representational similarity between the self and others.
	Loneliness and social connection	Caputi et al., 2017	Type of study: cognitive and self-report measures.<br>Participants: 326 children attending primary and middle schools.<br>Method: sociocognitive understanding task followed by self-report questionnaires on depressive symptoms and perceived loneliness.	Results: Correlational analyses showed that socio-cognitive understanding, depressive symptoms, and feelings of loneliness were significantly related. Mediation analyses revealed that feelings of loneliness mediated the effect of sociocognitive understanding on depressive symptoms, but only among girls. In boys, depressive symptoms were directly linked to sociocognitive skills.<br>Limits: relatively small sample size, which should be enlarged including also younger children.<br>Conclusions: In typically developing preadolescents, gender differences emerged in the role of loneliness in mediating the association between socio-cognitive understanding and depression
	Loneliness	Cacioppo et al., 2006	Type of study: telephone interview. (Study 1) + Longitudinal study with self-reported measures (Study 2).<br>Participants: 1945 participants aged 54 or above (Study 1), 212 participants between 50 and 67 years old.<br>Method: self-reported questionnaires assessing loneliness (3-item questions of social isolation, R-UCLA) and depressive symptoms (short and long form of the CES-D) + demographic information (e.g., education, marital status) + measures of psychosocial risk factors, such as perceived stress (Perceived Stress Scale - PSS), social support (Interpersonal Support Evaluation List - ISEL) and hostility (Cook-Medley Hostility Scale).	Results: In Study 1 higher levels of reported loneliness were associated with elevated levels of depressive symptoms. Moreover, when the individual psychosocial variables of perceived stress and social support were included as additional covariates, loneliness remained a significant predictor of depressive symptoms. The results of the hierarchical regression analyses using the more detailed measures in Study 2 replicated the major findings of Study 1. Longitudinal analyses revealed that loneliness in Year 1 predicted differences in depressive symptomatology in the subsequent years net of demographic variables, depressive symptoms, and other psychosocial variables in Year 1.<br>Limits: simplicity of statistical models (linear changes could not be distinguished from more complex forms) and relatively short

				duration panel.<br>Conclusions: loneliness and depressive symptoms have strong reciprocal influences in middle-aged and older adults.
	Loneliness	Cacioppo et al., 2000	Type of study: self-report and psychophysiological study.<br>Participants: 5% from our original sample of 2632 students previously assessed to determine their feelings of loneliness (UCLA loneliness scale and state and trait loneliness scores) and their living circumstances.<br>Method: Participants completed social and non-social speeches while Heart Rate (HR) was recorded, a dichotic listening task to test attentional control. The salivary cortisol levels across the day were collected. Participants completed the Pittsburgh Sleep Quality Index to assess sleep quality.	Results: Lonely individuals were characterized by greater anxiety, anger, and shyness, less sociability, less optimism, and poorer social skills, and they expressed stronger fears of negative evaluation. Lonely individuals were characterized by lower basal heart rate and lower heart rate reactivity than normal or embedded individuals. Lonely individuals failed to shift to an a priori predicted left-ear advantage in the focus on left-ear condition. Trait loneliness scores were positively and significantly correlated with mean cortisol levels. Moreover, lonely individuals slept less efficiently, took slightly longer to fall asleep, evidenced longer REM latency, and awoke more frequently during the night.<br>Limits: this paper provides a preliminary report of an ongoing study of the psychological and physiological differences between individuals differing in social embeddedness, thus participant sample, methods and analyses are not completely described.<br>Conclusions: Lonely individuals are more emotionally withdrawn when in a new social environment. This may be related to feelings of overwhelm because they have less control over the focus of their attention. Loneliness was associated with a range of altered physiological processes, including muted autonomic activation, elevated activation of the HPA axis and dysregulated sleep.
	Loneliness	Pressman et al., 2005	Type of study: biological measures (cortisol, immune response) + self-reported measures.<br>Participants: 83 University students.<br>Method: participants were immunized in conjunction with university-wide flu vaccination clinics. They participated to a 13-days ecological momentary assessment: Participants reported their current loneliness, stress, and affect four times daily (1, 4, 9, and 11 hours after waking up). They also reported their health practices once a day (how much they slept, smoked, consumed alcohol, and exercised). On days 2 through 6 of the protocol, participants gave salivary cortisol samples four times a day. Questionnaires were administered to assess loneliness (UCLA Loneliness Scale; Social Networks in Adult Life Questionnaire), stress and mood (Center for Epidemiological Studies Depression Scale - CESD-10);	Results: Loneliness was positively correlated with negative affect, psychological stress, and depressive symptoms and negatively correlated with positive affect. Loneliness was associated with baseline titers of A/New Caledonia.<br>Smaller social networks were associated with lower Ab production at both follow up points. Results not found any possible pathways linking social isolation to immune response.<br>Conclusions: social network size and loneliness were independently associated with the production of less Ab in response to one component of the influenza immunization in a young, healthy population.

			over the following 14 weeks, Ab [1]levels were assessed at baseline (day of immunization) and at 1- and 4-months post immunization.	
	Loneliness	Masi et al., 2011	Type of study: meta-analysis.<br>Sample: 50 articles were included.<br>Method: integrative meta-analysis of loneliness reduction interventions to quantify the effect of four primary intervention strategies: (a) improving social skills, (b) enhancing social support, (c) increasing opportunities for social contact, and (d) addressing maladaptive social cognition.<br>50 articles were included (12 single-group pre-post studies, 18 nonrandomized group comparison studies, and 20 randomized group comparison studies).	Results: Results revealed that single-group pre-post and nonrandomized comparison studies yielded larger mean effect sizes relative to randomized comparison studies. Among studies that used the latter design, the most successful interventions addressed maladaptive social cognition.<br>Limits: as for other systematic reviews, it is possible that the literature search failed to identify one or more interventions.<br>Conclusions: correcting maladaptive social cognition offers the best chance for reducing loneliness.
	Loneliness and Affective Touch	von Mohr et al., 2017	Type of study: self-reports.<br>Participants: 84 female young adults.<br>Method: Cyberball paradigm to manipulate ostracism (inclusion vs exclusion; within-participants) + tactile stimulation (slow-affective touch vs fast touch; between-participants) + self-report measures (Need-threat scale, Positive Affect and Negative Affect Schedule).	Results: across the groups, participants reported more need-threat in the Cyberball exclusion condition. While threat levels between the groups did not differ at baseline, there was a significant group difference in the exclusion condition, following touch manipulation. Participants that received slow touch post-ostracism reported less need-threat than those that received fast touch.<br>Limits: it remains possible that brush stroking may have missed essential mechanisms of skin-to-skin social support. Only women were included.<br>Conclusions: affective touch can lessen to a certain degree the distress caused by ostracism.
	Loneliness and Affective Touch	Heatley Tejada et al., 2020	Type of study: self-report and psychophysiological study.<br>Participants: 40 young adults (27 females, 13 males).<br>Method: participants completed questionnaires containing social network profiling, attachment style and loneliness measures (Multi-faceted Loneliness Inventory -IMSOL; short version of the UCLA loneliness scale) + HR recording. Participants in the experimental group received physical contact in the form of a light rub to the back of their hand, while the control group were asked to rub the oil themselves.	Results: Significant differences between groups (touch condition) and by relationship status in one of the loneliness scales that reflects perceptions of neglect. Regarding heart rate analysis, participants in the physical contact condition showed a significant decrease in heart rate.<br>Limits: Small sample size. Touch was brief and with apparent pragmatic purposes (rubbing the oil). Specific cultural setting (individualistic and low-contact culture).<br>Conclusions: physical contact has beneficial effects on loneliness and heart rate pointing to some important psychophysiological well-being implications.
**Social**	Virtual Reality	Slater et al., 2009	Type of study: IVR, self-reports.<br>Participants: 33 participants (15 women, 18 men).	Results: participants in RT reported a higher level of presence than those in RC. Between the baseline and experimental condition

**interactions in virtual reality**			Method: 3 minutes of virtual environment depicting a precipice, comparing a real-time recursive ray tracing (RT) that included shadows and reflections of their virtual body, and the same environment rendered with ray casting (RC), which did not include shadows and reflections. Participants completed a presence questionnaire immediately after their experience, and physiological responses (skin conductance and electrocardiogram) were recorded throughout.	people's arousal and orienting responses were on the average greater in the case of RT but not in the case of RC.<br>Limits: this study cannot disambiguate whether the changed responses were caused by the dynamic shadows and reflections rather than by the general improvement in visual quality that results from recursive ray tracing.<br>Conclusions: subjective presence was higher for the RT environment than for the RC one and that higher stress was reported in RT, suggesting that improved visual realism might enhance realistic behavioral response.
	Virtual Reality	Zopf et al., 2018	Type of study: IVR, behavioral, self-reports.<br>Participants: 30 neurotypical adults.<br>Method: manipulation of two aspects of movement feedback:<br>(a) form: participants' hand represented by either a virtual hand or sphere. (b) movement congruency: the virtual hand or sphere moved congruently or incongruently with the participant's real hand.<br>Measures of:<br>(1) embodiment: self-report ratings of rubber hand ownership<br>(2) implicit agency. intentional binding task (participants judged the interval between pressing a button and a subsequent tone)<br>(3) explicit agency: Sense of Agency Rating Scale.	Results: Both form and movement congruency significantly influenced embodiment. Only movement congruency influenced intentional binding, which was increased for congruent than incongruent feedback.<br>Limits: not reported.<br>Conclusions: both embodiment and agency depend on comparisons across sensorimotor signals, but they are influenced by distinct factors. Implicit (but not explicit) agency over one's own movements depends on feedback congruency, whereas bodily realism is not fundamental.
	Virtual Reality	Gall, et al., 2021	Type of study: IVR, self-reports.<br>Participants: 21 female young adults.<br>Method: Participants saw a virtual representation of their right hand. Visuotactile stimulation was used to induce the illusion (synchronous vs asynchronous conditions) + presentation of 20 pictures from the international affective picture system. Participants self-assessed valence, arousal, and dominance on self-assessment manikin scales and a one-item presence scale.	Results: in the synchronous stimulation condition participants reported significantly higher virtual body ownership and agency. They also reported significantly higher arousal and dominance (positive valence translates to high ratings and negative valence translates to low ratings).<br>Limits: the method does not allow to identify which subcomponent of embodiment drives the observed effect (virtual body ownership, agency, or presence). Small effect size. Only three items have been used to assess virtual embodiment and no physiological measures.<br>Conclusions: Virtual embodiment intensifies emotional response to virtual stimuli.
	Virtual Reality	Dewe et al., 2021	Type of study: IVR, self-reports.<br>Participants: 197 children aged 4-14.<br>Method: children interacted with the virtual environment	Results: participants embodied both virtual forms to some degree, in synchronous conditions. Only the synchronous hand was perceived as part of the body, rather than a tool. Haptic feedback

			through a virtual hand which moved synchronously or asynchronously with their actual movements.<br>(Experiment 1) participants additionally felt haptic feedback either congruently, delayed or not at all.<br>(Experiment 2) participants used either a virtual hand or non-human virtual block.<br>Measures: self-reported embodiment (ownership and agency over, and location of the virtual hand).	did not influence embodiment.<br>Limits: use of short (limited sensitivity) self-reports, which can be biased, especially with children (confounding mechanisms such as individual verbal comprehension). Mainly male, right-handed sample.<br>Conclusions: overall dominance of visuomotor synchrony for children's own-body representation.
	Virtual Reality	Segovia & Bailenson, 2009	Type of study: IVR and memory study (narrative + memory interview).<br>Participants: 55 preschool and elementary children.<br>Method: children were randomly assigned to 1 of 4 memory prompt conditions (just sit and wait for 1-minute, mental imagery, virtual simulation of another child, or virtual simulation of self). Regardless, the condition, the researcher read the false event narrative to the child. Each child was interviewed 3 different times: before immediately after and 5 days after the memory prompt. Children's memories were coded according to four categories: no memory; no memory but trying to recall; partial memory, but little detail; memory with full detail.	Results: For preschool children, the false memory scores were higher after the experimental treatment than in the baseline interview, but the main effect of memory prompt was not significant.<br>For elementary children, the false memory scores were highest just after the false memory prompt and the main effect of the memory prompt was significant. Additional tests of simple effects revealed the mental imagery and virtual self-conditions elicited significantly more false memories.<br>Limits: small age range. This design doesn't allow to separate the effect of the narrative from the other conditions (each condition included the reading of the narrative).<br>Conclusions: for elementary children, the mental imagery and virtual self-conditions caused significantly more false memories.
	Virtual Reality and Affective Touch	de Jong et al., 2017	Type of study: IVR full body illusion.<br>Participants: 20 female young adults (Study 1) 19 female young adults (Study 2).<br>Method: virtual reality full body illusion. Visuo-tactile stimulation was performed either synchronously or asynchronously (control condition) on the participants' abdomen at two different velocities (slow/affective touch vs fast condition) + EPTQ and EQ questionnaires (Study 2).	Results: Study 1 revealed that affective touch (slow condition) was rated as more positive than fast touch. Moreover, affective touch resulted in higher levels of body ownership and agency.<br>Study 2 revealed that participants rated the synchronous conditions and the slow touch as more pleasant. Both affective and non-affective touch elicited an equally strong subjective experience of the illusion in terms of ownership over the avatar.<br>Limits: Small sample size. Only female participants.<br>Conclusions: given the inconsistency of results from Study 1 and study 2, it remains unclear whether affective touch enhances the full-body illusion.
	Virtual Reality and Affective Touch	Carey et al., 2021	Type of study: IVR full body illusion.<br>Participants: 38 (Study 1) + 39 (Study 2) female young adults.<br>Method: full-body illusion with a life-size female mannequin. The experiment used a 2 (stroking velocity: affective/slow vs. non-affective/fast) × 2 (stroking	Results: study 1 and 2 revealed a main effect of stroking velocity and synchrony on subjective pleasantness and a greater embodiment change following the synchronous condition compared to the asynchronous one. No significant correlations emerged between subthreshold ED psychopathology (EDE-Q) and experimental measures (pleasantness, embodiment, touch difference score).

			synchrony: synchronous vs. asynchronous) within-subjects design. Study 1 manipulates synchrony in terms of temporal matching, while Study 2 in terms of spatial congruency of visuo-tactile stimulation. Subjective pleasantness of stroking (VAS scale) and Subjective embodiments (Embodiment Questionnaire - EQ) were measured + Eating Disorder Examination Questionnaire (EDE-Q).	Limits: only female participants and only subthreshold measure of eating disorders.<br>Conclusions: affective touch is perceived as more pleasant than non-affective touch. However, such effects of stroking velocity during multisensory integration did not modulate the subjective embodiment towards a whole mannequin body within the full body illusion.
	Virtual Reality	von der Pütten et al., 2010	Type of study: IVR, human-computer interaction.<br>Participants: 83 participants (42 women and 41 men).<br>Method: human-computer interaction 2 × 2 design: agency (agent vs avatar) and behavioral realism (showing feedback behavior vs showing no behavior). Rapport Agent to establish coordination between the interactants. After the interaction, self-reported experience of social presence, self-reported rapport, and participants' emotional state (PANAS) + analyses of verbal behavior.	Results: main effect for agency: participants who thought they were interacting with an artificial agent experienced more negative feelings. Social presence was more intense, and the total amount of words was twice in the condition with high behavioral realism.<br>Limits: complex meaningful human interaction are harder to implement and synthesize.<br>Conclusions: the belief of interacting with either an avatar or an agent barely resulted in differences regarding the evaluation of the virtual character or behavioral reactions, whereas higher behavioral realism affected both.
	Virtual Reality and Affective Touch	Hoppe et al., 2020	Type of study: IVR, human-virtual character tactile interaction.<br>Participants: 19 participants (6 women, 13 men).<br>Method: artificial hand to apply social touch during a virtual social interaction. 2x2 design: touch (touch vs no-touch) and virtual character (virtual agent vs avatar controlled by the experimenter). After the interaction, self-reported measures on co-presence, embarrassment, likeability, and perceived agency.	Results: main effect of the factor Entity, indicating that participants were able to discriminate between agents and avatars. The introduction of touch significantly increased the ratings regarding Perceived Agency, the felt Co-Presence and Embarrassment.<br>Limits: participants with different experience with IVR.<br>Conclusions: social touch is effective to blur the boundary between computer- and human-controlled virtual characters and contributes to experiences that closely resemble human-to-human interactions.
	Virtual Reality	Kroczek e al., 2020	Type of study: IVR, self-reported and psychophysiological measures.<br>Participants: 36 young adults (18 women, 18 men).<br>Method: Real-time social interaction with virtual agents. Questionnaires were used to assess social anxiety (Social Phobia Inventory - SPIN), presence (IPQ), state and trait anxiety (STAI), and demographic information. Using the median split of SPIN, participants were assigned into two groups: Low social-anxious participants (LSA) and high social-anxious participants (HSA). As a behavioral measurement, the distance between participant and each of the agents was recorded. Physiological measurements	Results: social interactions at 1 and 1.5 m were rated as more arousing and less pleasant than longer distances greater than 2 m and elicited an increase in SCR. Social interactions at a short distance were rated as less realistic compared to longer distances, but this effect was only present in high social-anxious participants. The HSA group showed increased arousal and rated interactions as less pleasant compared to the LSA group. Moreover, there was increased retraction away from the virtual agent at a short interaction distance in the HSA group.<br>Limits: it is quite challenging to provide highly realistic social interactions in VR. Individually preferred interpersonal distance was not assessed. The analysis of social anxiety based on a median

			included ECG, EDA, and EMG.	split combined with a relatively small sample size brings some limitations with respect to statistical power.<br>Conclusions: in IVR, closer distances were rated as more arousing, less pleasant, and less natural than longer distances and this effect was significantly modulated by social anxiety scores.
**Impact of interpersonal virtual interactions on loneliness**	Loneliness and Virtual communicatio n	Coget et al., 2002	Type of study: telephone survey.<br>Participants: 2096 randomly chosen individuals (both Internet users and nonusers) aged 12 and above.<br>Method: measure of loneliness (3 items from the UCLA loneliness scale) and social isolation (7 items) + questions of Internet use + measure of digital and face-to-face socializing (number of friends, time spent in socializing).	Results: among Internet users, time spent weekly on the Internet was associated with a higher probability of socializing online. Time spent weekly online, experience and digital socializing were not associated with a reduced number of face-to-face friends. Digital socializing was associated with increased loneliness, controlling for other possible effects of the Internet.<br>Limits: one-time correlational design, which does not allow for causal inferences. The measure of loneliness did not include the full set of items of the UCLA loneliness scale. Given the fast pace at which the Internet is spreading and changing, any result of studies done on its social impact is at risk of becoming obsolete.<br>Conclusions: overall, the impact of the Internet on loneliness is unclear and complex. No effects of the Internet on people's social networks are found, but there is a mild positive effect of the Internet on people's loneliness.
	Loneliness and Virtual communicatio n	Daniel et al., 2018	Type of study: iterative theory-building approach, semi-structured interview.<br>Participants: 23 participants: step 1) 5 home-based workers known personally to match study requirements; step 2) they were asked to identify others with online businesses (7 participants); step 3) using social media, other 11 participants highly congruent with the population of interest were recruited.<br>Method: inductive methodology and qualitative design involving in-depth interviewing. Transcription of all semi-structured interviews were coded thematically using Nvivo software.	Results: participants generally viewed their present careers as positive, life-affirming choices. Some missed face-to-face aspects of work contacts. Participants described social isolation feelings, with few face-to-face, informal interactions with people generally, not just with former work-based colleagues. Participants described contrasting negative feelings of loneliness with positive ones of reflective solitude.<br>Limits: this approach can't provide quantitative data.<br>Conclusions: home-based knowledge-worker is multi-layered, complex, and paradoxical. It involves the need for autonomy and creative management of time and place, and a sense of unease and loneliness relieved only through contact with others.
	Internet/social media use and well-being, Loneliness and Virtual communicatio n	Orben & Przybylski, 2019	Type of study: correlational study across large-scale social datasets.<br>Participants: 355,358 from 3 large-scale datasets—Monitoring the Future (MTF), Youth Risk and Behavior Survey (YRBS) and Millennium Cohort Study (MCS), predominantly 12- to 18-year-old adolescents surveyed between the years 2007 and 2016.	Results: the SCAs showed that there is a small negative association between technology use and well-being, however it is not possible to make many analytical statistical inferences because the specifications are not part of the same model and are not independent.<br>Limits: the high likelihood of unaccounted factors affecting both technology use and well-being. Many measures used were also of

			Method: specification curve analysis (SCA) across datasets. Measures of both digital technology use and psychological well-being. Sociodemographic factors and maternal characteristics were included as covariates. Specifications were identified (step 1), implemented (step 2) and compared (step 3) to compare the effects of digital technology to those of other activities on adolescents' psychological well-being.	low quality, non-normal, heterogenous or outdated, limiting the generalizability of the study's inferences. Moreover, simple linear regressions were used in this study, overlooking the fact that the relationship of interest is probably more complex, non-linear, or hierarchical.<br>Conclusions: the association between digital technology use and adolescent well-being is negative but small, explaining 0.4% of the variation in well-being.
	Loneliness and Virtual communicatio n	Cauberghe et al., 2021	Type of study: survey study.<br>Participants: 2,165 adolescents (13–19 years old; 66.6% women, 34.4% men) recruited in April 2020.<br>Method: structural equation modeling approach. Happiness was measured by 3 items of the Center of Epidemiological Studies-Depression Scale; anxiety feelings over the last month were measured by the General Anxiety Disorder Scale (GAP-7); loneliness was measured by a 6-item scale (RULS-6). Finally, the Brief-Coping Scale was adapted to measure how participants use social media to cope with the COVID-19 situation.	Results: higher feelings of anxiety and loneliness were both associated with lower feelings of happiness. Anxiety was significantly related to active (social media) and social coping, while loneliness was positively associated only with social coping. Neither loneliness nor anxiety was associated with humorous coping. Active coping and humorous coping were significantly positively related to happiness, while social coping was not.<br>Limits: self-selection bias may be present in the sample. Further research should use scales developed for the COVID-19 situation. Cross-sectional study does not allow to examine causality among the variables.<br>Conclusions: participants who were feeling lonely were more inclined to use social media to cope with lacking social contact. However, this coping strategy was not significantly related to their happiness feelings.
	Loneliness and Virtual communicatio n	Primack et al., 2017	Type of study: online survey.<br>Participants: 1,787 young adults (50.3% women; 49.7% men).<br>Method: social media use was assessed using both time and frequency associated with use of 11 social media platforms. Social isolation was measured with PROMIS social isolation scale. Logistic regression was used to assess associations between social media use and social isolation while controlling for eight covariates (sex, age, race/ethnicity, education, household income, Census region, metropolitan area, and Internet access).	Results: in fully adjusted multivariable models that included survey weights, participants in the highest quartile for social media frequency and time showed both higher feelings of social isolation.<br>Limits: low-intensive measures of social media exposure. Self-reported measures are subject to recall and social desirability biases. Sample included only young adults.<br>Conclusions: young adults with high social media use seem to feel more socially isolated.
	Loneliness and Virtual communicatio n	Pittman, 2015	Type of study: online survey.<br>Participants: 432 undergraduates (75% women, 25% men).<br>Method: the social media platforms<br>examined in this study are Facebook, Twitter, and Instagram. Participants were asked questions about social	Results: respondents with positive attitudes about social media were significantly less likely to be lonely. This correlation was only found for Twitter and Instagram, not Facebook. There was no significant difference in loneliness between those who create social media content and those who consume it.

			media attitude, social media behavior, and loneliness (UCLA Loneliness Scale).	Limits: only self-reported data, sample non representative of entire population (only undergraduates in a single city). Individual variables should be considered.<br>Conclusions: students' affinity for and use of Twitter and Instagram was associated with less self-reported loneliness.
	Loneliness and Virtual communicatio n	Shillair et al., 2015	Type of study: longitudinal study (internet training).<br>Participants: 306 old adults (80% women, 20% men) at 19 assisted and independent living communities.<br>Method: mediation moderation regression analysis. 3 conditions: Internet training, attention control (placebo), and true control (no training). Surveys at baseline (T1) and at the end of an 8-week training intervention (T2) + follow up at 3 months (T3), 6 months (T4), and 12 months (T5). Demographics were measured for age, race, and education. Socioemotional variables included social support (MOS social support scale), loneliness (UCLA loneliness scale), and life satisfaction questions; attitude towards the Internet was measured as a moderator.	Results: at all time points loneliness has a significant negative effect and social support has a positive effect on life satisfaction. At T1, T4 and T5 the interaction effect of social support and Internet attitude is positive and significant.<br>Limits: accurately measuring levels of life satisfaction and loneliness are often difficult with older adults. Outside situations, beyond the scope of this research may also be contributing to the sense of loneliness or life satisfaction of the participants.<br>Conclusions: older adults who felt confident in being able to use Internet technologies to communicate with others, there was a moderating effect on the life satisfaction levels.
	Loneliness and Virtual communicatio n	Pittman, 2018	Type of study: self-reports.<br>Participants: 352 undergraduate students (75.4% women, 24.6% men).<br>Method: Loneliness (UCLA loneliness scale), happiness (4-items), perceived intimacy (3 items) and frequency of social media use were measured.	Results: loneliness predicted a decrease in perceived intimacy of social media, while happiness predicted an increase in perceived intimacy of social media. Social media use was a significant predictor of perceived intimacy and perceived intimacy was in turn a significant predictor of loneliness and happiness (mediation).<br>Limits: sample only included college students. Cross-sectional study that only captured users' responses at a single moment.<br>Conclusions: social media have potential for reducing loneliness and increasing happiness, but only in the degree to which their use is perceived as intimate.
	Loneliness and Virtual communicatio n	Williams et al., 2000	Type of study: online experiment, self-reports.<br>Participants: 1,486 participants from 62 countries.<br>Method: participants were asked to use mental visualization while playing a virtual tossing game with two others (who were computer generated). The quantity of ostracism (i.e., the probability that participants would receive the disc) was manipulated. Post-test self-reported measures of needs threatened and mood.	Results: less probability of receiving the disc increased participants' negative feelings, reduced their perception of control and belonging.<br>Limits: self-reports.<br>Conclusions: the ostracism paradigm is effective also within internet-mediated interactions.
	Loneliness, Social	von Mohr et al., 2017	Type of study: behavioral, self-reported measures.<br>Participants: 84 women.	Results: the provision of slow-affective, as compared to fast-neutral, touch led to a specific decrease in feelings of social

	connection, and Virtual communicatio n		Method: social exclusion paradigm in 2 phases:<br>a) Cyberball-inclusion game for approximately 2–3 minutes. All players received an equal number of ball-tosses. Upon completion, participants rated twenty-items of needs that are often threatened by ostracism.<br>b) Cyberball-Exclusion game for 2–3 minutes; participants received the ball 2 initial times, while they were excluded in the remaining ball-tosses. Upon completion, participants were blindfolded and received 70-seconds brushing in either: CT-optimal speed (3 cm/s; slow touch group) or non-CT-optimal speed (18 cm/s; fast touch group). Following tactile stimulation, participants filled out the main measure of ostracism (Need-threat scale).	exclusion, beyond general mood effects.<br>Limits: potential individual differences and higher order top-down processes were not accounted for. Only female participants.<br>Conclusions: affective touch, particularly in the context of social separation or rejection might have a regulatory function, thus reducing the impact of negative social experiences.
**Bringing interpersonal affective touch into virtual reality**	Interpersonal Affective Touch and Virtual Reality	Thomas & Glowacki, 2018	Type of study: multi-user IVR in shared physical environment, workshop, qualitative self-reported measures.<br>Participants: 20 adults.<br>Method: 40-minute practical session into the virtual environment, followed by a 20-minute group discussion. Guided exploration with open or closed eyes, with or without touching things and other participants, in solo or social conditions.<br>Qualitative self-reported measures of bodily experience in IVR.	Results: people experiencing their bodies as felt but not seen.<br>Limits: the project comes from the arts field and qualitative measures are hard to interpret scientifically.<br>Conclusions: open speculations about how we can leverage IVR's potential to promote new modes of one's own body perception and knowledge.
	Interpersonal Affective Touch and Virtual Reality	Ahmed et al., 2016	Type of study: IVR, self-reported.<br>Participants: 17 university students.<br>Method: in response to the participants' reaching action in the space between themselves and a virtual agent, the agent displayed one out of five types of emotional expression (happiness, sadness, anger, fear, neutral). The animated agent then delivered interpersonal touch to the participant's hand. Touch on the real hand was simulated using two types of vibrotactile actuators and two types of force feedback mechanisms. Self-reports of the touch perception (naturalness and intensity) co-presence and emotional interdependence were obtained after each trial.	Results: force feedback actuators were rated as more natural and resulted in greater emotional interdependence and co-presence than vibrotactile actuators. Happy facial expressions elicited higher emotional interdependence and co-presence than neutral faces.<br>Limits: technical limitations are discussed. Self-reports can be biased.<br>Conclusions: multimodal affective communication is better simulated through force feedback than vibrotactile devices, thus potentially increasing social connection between users.
	Interpersonal Affective Touch and Virtual Reality	Bailenson & Yee, 2008	Type of study: IVR, behavioral.<br>Participants: 40 undergraduate students.<br>Method: participants were told to use the haptic device to clean "dirt spots" off some virtual objects or human	Results: participants used less force when touching virtual people than non-human objects and touched faces with less force than torso areas. Male digital partners were touched with more force than female partners.

			characters. Measure of force applied.	Limits: the lack of direct evidence for the association between force adjustments and perceived co-presence reduces the authors' conclusions to speculation to be tested further.<br>Conclusions: interpretations of findings as an implicit measure of co-presence.
	Interpersonal Affective Touch and Virtual Reality	Bailenson et al., 2007	Type of study: haptics, kinematics, self-reports.<br>Participants: 2 independent groups of 16 undergraduate students (experiment 1 and 2), 32 undergraduate students (experiment 3).<br>Method:<br>Experiment 1: participants utilized a force-feedback joystick to express seven emotions. measure of various dimensions of the forces generated and subjective ratings of the difficulty of expressing those emotions.<br>Experiment 2: a separate group of participants attempted to recognize the recordings of emotions generated in experiment 1.<br>Experiment 3: pairs of participants attempted to communicate the seven emotions using physical handshakes through a doorway covered by a curtain.	Results:<br>Experiment 1: handshaking behaviors varied when different emotions were being expressed and can be distinguished from their kinematics.<br>Experiment 2: above-chance emotion recognition accuracy.<br>Experiment 3: greater emotion recognition accuracy than experiment 2.<br>Limits: basic haptic device. The task was not very naturalistic (on demand emotions), and targeted pure tactile aspects, with no other verbal and non-verbal cues that are, however, intrinsically linked to tactile exchanges.<br>Conclusions: to some extent, people can recognize emotions via mediated touch, but not as accurately as when expressing emotions through skin-to-skin handshakes.
	Interpersonal Affective Touch and Virtual Reality	Haritaipan et al., 2018	Type of study: video-call, haptics, behavioral, self-reports.<br>Participants: 8 Chinese couples in romantic relationships.<br>Method:<br>Implementation of "sender device" for the person giving the massage and a "receiver device" for the person receiving the massage. Couples engaged in remote video calls either with or without using massage-assisted devices. They managed conversations about happy, sad, or angry topics.	Results: Significant increase in total massage time and average force per finger, from positive conversation to negative conversation. Increase in self-reported emotional connection from video call only to video call and massage. 87.5% of participants suggested that they would like to use the device in long-distance communication with their parents.<br>Limits: small sample and cultural specificity limit generalizability of findings. Technical aspects are discussed.<br>Conclusions: mediated audio-visual and haptic communication interplay. Interpersonal touch can be implemented into remote communications through haptic devices, thus supporting social relationships.
**Disconnection from the bodily self: Anorexia Nervosa**	Anorexia, Social connection and Loneliness	Schlegl et al., 2020	Type of study: survey.<br>Participants: 159 female patients with AN who have been discharged from treatment.<br>Method: online survey on contact history with COVID-19, changes in eating disorder symptoms and other psychological aspects, health care utilization, and strategies patients employed to cope during the pandemic.	Results: 70% of patients reported that both eating symptoms and loneliness increased during the pandemic; access to therapies decreased.<br>Limits: only female sample, self-reports can be biased, correlational study with no clues on the direction of associations among variables, no control group.<br>Conclusions: interventions addressing symptoms of depression and

				anxiety, intolerance of uncertainty might help AN patients manage their eating disorder symptoms.
	Anorexia and Affective Touch	Crucianelli et al., 2016	Type of study: self-reports.<br>Participants: 25 AN patients and 30 controls.<br>Method: pleasantness ratings of C-Tactile (CT) afferents-optimal (3 cm/s) and non-optimal (18 cm/s) touch (forearm stroking), while simultaneously displaying images of faces showing rejecting, neutral and accepting expressions.	Results: CT-optimal touch, but not CT-non-optimal touch, elicited significant lower pleasantness ratings in AN patients compared to controls. Pleasantness ratings were modulated by facial expressions in both groups in a similar fashion.<br>Limits: absent measure of potential confounding aspects, such as social cognition difficulties, (i.e., face recognition) and attentional biases of participants with AN.<br>Conclusions: individuals with AN have a disordered, CT-based affective touch system. This impairment may be linked to reduced interoceptive perception and atypical body representation.
	Anorexia and Affective Touch	Davidovic et al., 2018	Type of study: fMRI, self-reports, stimulation procedure.<br>Participants: 25 female participants with AN, 25 female controls (HC).<br>Method: BDI, EHI (handedness inventory).<br>The procedure had three conditions: skin stroking condition, gently touching the skin but not moving condition, and brush rest condition with static skin indentation. Participants were instructed to lie still with their eyes closed, and to focus on how pleasant/unpleasant the tactile sensation felt.	Results: AN patients rated skin stroking as less pleasant than HC, and showed less activation (left caudate nucleus, bilateral frontal pole, right temporal pole) during skin stroking than skin indentation.<br>AN patients showed less bilateral lateral occipital cortex (LOC) activation for skin stroking than HC.<br>Limits: psychiatric comorbidity not assessed, confounding effects of anhedonia.<br>Conclusions: AN patients showed less activation of different brain areas during skin stroking. The abnormal LOC response to skin stroking might be related to disturbed body image perception.
	Anorexia and Affective Touch	Bischoff-Grethe et al., 2018;	Type of study: fMRI, stimulation procedure, self-reports.<br>Participants:18 female participants recovered from anorexia nervosa (RAN), 26 healthy women (CW).<br>Method: soft touch continuous performance task.<br>Participants are presented on the screen with an arrow (pointing left vs. right) whose background anticipates: baseline (no stimulus was expected), soft touch or brushing on the forearm or palm. Participants were asked to press the left or right button corresponding to the direction of the arrow.	Results: Both groups showed a greater brain response during the soft touch stimulation rather than in the anticipation phase (bilateral insula and dorsal striatum).<br>In the right ventral mid-insula, RAN had a lower BOLD response during anticipation, but a greater BOLD response during soft touch than CW.<br>Both groups showed a greater response during soft touch compared to anticipation (right ventral mid-insula and superior temporal gyrus), but this difference was more pronounced in the RAN group than the CW.<br>Limits: small, only female sample; it is not possible to determine whether findings are related to some AN core traits or are a consequence of malnutrition.<br>Conclusions: AN is associated with altered neural signals that predict and interpret pleasant tactile stimuli.

	Anorexia and Affective Touch	Crucianelli et al., 2021	Type of study: stimulation procedure, self-reports.<br>Participants: 27 female participants with AN, 24 female participants recovered from AN (rAN), 30 healthy controls (HC).<br>Method: self-reports: interoceptive awareness (from EDI-2); interoceptive sensibility (BAQ), alexithymia (TAS-20).<br>Procedure: 1) imagined tactile pleasantness procedure: participants answer four hypothetical questions about imagined touch.<br>2) affective touch protocol: experimenter delivers touch at five different speeds (two optimal for CT, one borderline and two suboptimal for CT). After each trial participants rated pleasantness.	Results: AN and rAN groups anticipated and perceived affective touch as overall less pleasant compared to HCs; reduced pleasantness was not specific to the CT-optimal stroking velocities since it was also for suboptimal stroking.<br>Instead, differences in the perception of CT-optimal touch were predicted by differences in top-down beliefs, alexithymia and interoceptive sensibility.<br>Limits: relatively small, only female sample.<br>Conclusions: anticipation and perception of pleasant touch is reduced in patients with acute AN and those who have recovered from AN, compared to HCs. Such tactile anhedonia is not explained by deficits in the CT-afferent system.
	Anorexia and Affective Touch	Hart et al., 2001	Type of study: Massage therapy, self-reports, biological measures.<br>Participants:19 female participants diagnosed with AN.<br>Method: participants randomly assigned to massage therapy (N=10) or a standard treatment control group. Massage therapy group received a 30-minute massage, two days per week for five weeks, for a total of ten massages.<br>Pre- and post- treatment measures:<br>STAI (anxiety inventory); POMS (profile of mood states); CES-D (depression scale); EDI (eating disorder inventory); Saliva Cortisol samples (stress hormone levels); Urine samples (catecholamines, cortisol, dopamine).	Results: massage therapy reduced anxiety, improved mood, lowered cortisol levels. After the 5-week treatment period the massage therapy group had: lower scores on EDI, significant increase of dopamine and norepinephrine levels.<br>Limits: no data gathered on nutrition, only female and small sample, attribution of increased dopamine levels to massage was speculative.<br>Conclusions: massage therapy attenuated anxiety, eating disorder symptoms, poor body image, and biochemical abnormalities for women diagnosed with AN.
	Anorexia and Virtual Reality	Serino et al., 2019	Type of study: IVR, self-reports.<br>Participants: single-case study (one woman with AN).<br>Method: treatment protocol that included three sessions of a body swapping illusion (i.e., the experimental induction of being the owner of a virtual body because of a visuotactile stimulation - synchronous vs asynchronous condition). Pre- and post-treatment + 1-year follow-up measurements: self-reported body-size estimation, level of embodiment of the virtual body.	Results: successful embodiment only pre-treatment, in both synchronous and asynchronous conditions. Pre-treatment body size overestimation. Decreased over-estimation at follow-up.<br>Limits: results based on a single patient, mixed findings on the link between embodiment and body size estimation, improvements at follow-up not clearly attributable to VR stimulation.<br>Conclusions: VR full body illusion is promising to monitor and promote changes of bodily perceptions.
	Anorexia and Virtual Reality	Keizer et al., 2016	Type of study: IVR, self-reports.<br>Participants: 30 AN patients, 29 controls.<br>Method: VR full body illusion (FBI) (synchronous and asynchronous visuotactile stimulation). Post-test and 3-hours follow-up measurements: self-reported body size	Results: AN patients and controls had an equally strong experience with the FBI. AN patients reported pre-test overestimation, which decreased after the FBI was induced and (to some extent) at follow-up.<br>Limits: only female samples, self-reports can be biased or not

			estimation, level of embodiment of the virtual body.	sensitive measures of subtle changes in body perception.<br>Conclusions: body size estimation of patients with AN can be changed through VR body illusions.
**Self-other disconnection: the case of Autism**	Autism, Social connection and Loneliness	Schiltz et al., 2021	Type of study: self-reports.<br>Participants: 69 young adults with ASD.<br>Method: self-report questionnaires assessing social contact (Friendship Questionnaire), autism features (Autism Quotient), mental health (Liebowitz Social Anxiety Scale, Social Phobia Inventory, Beck Depression Inventory), and loneliness (Social and Emotional Loneliness Scale for Adults).	Results: positive associations between autism features, loneliness, social anxiety, and depression. More social contact was related to less loneliness and less social anxiety. Mediation analyses indicated significant indirect effects of social contact and autism features on mental health through social loneliness.<br>Limits: no control group, the cross-sectional design limits the possibility to draw causal conclusions. The sample was small and homogeneous, thus limiting generalization of findings. Self-reports can be biased.<br>Conclusions: the link from social engagement and autism features to social anxiety and depression symptoms could be mostly explained by loneliness.
	Autism and Affective Touch	Kaiser et al., 2016	Type of study: fMRI.<br>Participants: 19 children and adolescents with ASD (16 male participants), 19 TD children and adolescents.<br>Method: participants received brushing to the right palm (non-CT targeted) or forearm (CT targeted). These two conditions were alternating., with a speed of 8cm/s (optimal for CT afferents).<br>Tests: Autism Diagnostic Observation Schedule (ADOS), Calibrated Severity Score (CSS).	Results: TD children and adolescents exhibited Arm > Palm activity in the bilateral insular cortex extending into the vlPFC and the temporal poles as well as the right pSTS, right amygdala, and right fusiform gyrus (FG). Participants with ASD lacked Arm > Palm activity in the right pSTS, TPJ and IPL, right FG, right amygdala, bilateral insula and vlPFC.<br>ASD and TD groups displayed distinct differential responses to the (Arm > Palm) and (Palm > Arm) contrasts: TD group uniquely exhibited an enhanced response to CT-targeted Arm > Palm touch, while the ASD group uniquely exhibited an enhanced response to non-CT-targeted Palm > Arm touch.<br>Touch was negatively correlated with ADOS and CSS scores in the mPFC, suggesting that altered processing of gentle touch, regardless of the touch location, is associated with ASD symptom severity.<br>Limits: imbalance between males and females in the ASD and TD groups, relatively small sample size<br>Conclusions: people with ASD do not manifest targeted activation, in response to affective touch, in some brain regions known to process social-emotional information.
	Autism and Affective Touch	Voos et al., 2013	Type of study: fMRI.<br>Participants: 19 healthy adults (12 women).<br>Method: before and during the fMRI scanning the procedure consisted of alternating blocks of slow (optimal	Results: negative correlations between the AQ and neural response to CT-targeted affective touch; subjects with more autistic traits show a lower differential response to affective touch in right OFC and right STS regions. Greater functional connectivity between the

			CT stimulation) and fast (suboptimal CT stimulation) brushing to the forearm.<br>Participants rated the pleasantness for each type of touch on a Likert scale, completed the Social Touch Questionnaire and AQ to measure autistic traits.	mPFC and regions in the right amygdala, left amygdala and left insula during the slow touch condition.<br>Limits: small sample, self-reports can be biased.<br>Conclusions: key nodes of the social brain are specifically involved in processing affective touch by CT-afferents and the neural response to affective touch has a relationship with the presence of autistic traits.
	Autism and Affective Touch	Silva et al., 2015	Type of study: replication study of treatment protocol.<br>Participants: 103 preschool children with ASD of which 53 in the treatment group, 48 in the control group (age range = 2:5).<br>Method: multisite, randomized, single-blind, controlled trial of the QST Dual massage.<br>Treatment group received 5 months of daily massage from parents.<br>Outcome measures: CARS2, PLS-5, Vineland-II, abc, SSC, APSI, Beach center Family-Professional Partnership Scale, Fidelity and Social Validation Testing.	Results: treatment group showed significant improvements on all measures. Overall treatment effect in reducing the autism severity as measured by the CARS and ABC. Effect size was in the medium to large range. Significant treatment effect on expressive language, sensory profile, and self-regulation.<br>Parents of children with autism experienced significant reduction in stress. Treatment was effective for both low- and high-functioning children.<br>Limits: not reported.<br>Conclusions: the QST Dual program for autism directed at tactile abnormalities was effective in decreasing severity of sensory, behavioral, and language components of autism as well as overall severity of autism. The intervention works by decreasing tactile and other sensory abnormalities.
	Autism and Virtual Reality	Mul et al., 2019	Type of study: IVR, behavioral, self-reports<br>Participants: 22 adults with ASD and 29 neurotypical adults.<br>Method: VR full body illusion (visuotactile synchronous vs asynchronous stimulation). Measures: drift in perceived self-location, peripersonal space (audiotactile reaction time task). Self-report questionnaires for autistic traits (AQ), empathy (QCAE), cognitive empathy (QCAEcog), alexithymia (TAS20) and the MAIA subscale of attention regulation.	Results: participants with Autism are less susceptible to the full body illusion (not demonstrating the illusory self-location drift), with no differences between synchronous and asynchronous conditions. This is correlated with severity of autistic traits and reduced empathy scores. Patients also show smaller peripersonal space.<br>Limits: self-reports might be unreliable. Sample is limited.<br>Conclusions: less flexible representation of the bodily self in participants with Autism, potentially due to differences in multisensory integration, and linked to deficits in social functioning.
	Autism and Virtual Reality	Simões et al., 2020	Type of study: IVR, behavioral.<br>Participants: 25 adults with ASD and 23 controls.<br>Method: stop-distance paradigm: participants stop themselves or the partner as soon as they reach the perceived interpersonal comfort distance (IPD). Task managed in real or equivalent VR environments.	Results: higher variability in the comfort IPD in the ASD group. Individuals with ASD choose similar IPD in virtual and real worlds, while controls show rescaling.<br>Limits: small sample; absent evaluation of individual differences that might contribute to explain findings.<br>Conclusions: people with ASD might similarly interact with others

				in real and virtual environments.
	Autism, Affective Touch and Virtual Reality	Vaucelle et al., 2009	Type of study: design of haptic interfaces for therapy.<br>Participants: none.<br>Method: design and building of four haptic devices that rely on vibrotactile, pneumatic and heat pump actuation, sensor-based and mechanically driven technologies.	Results: implementation of Touch Me, Squeeze Me, Hurt Me, Cool Me Down.<br>Limits: devices have not been tested with target users.<br>Conclusions: these devices need to be considered for clinical trials.
	Autism, Affective Touch and Virtual Reality	Tang et al, 2014	Type of study: design of haptic interfaces for therapy.<br>Participants: 4 informal users.<br>Method: design and development of a tactile sleeve to help ASD patients manage hypersensitivities to human contact through virtual experiences of being touched.	Results: participants were able to recognize virtual gestures transmitted through the device. One participant complained that the vibrotactile stimulations were sometimes excessive and uncomfortable.<br>Limits: the device has not been tested with target users.<br>Conclusions: the device needs to be improved and considered for clinical trials.
**Disconnected from the other: interpersonal violence**	Interpersonal Violence, Social connection and Loneliness	Eslea et al., 2004	Type of study: survey.<br>Participants: 48.000 children.<br>Method: children from China, England, Ireland, Italy, Japan, Portugal, and Spain completed various translations of the Olweus Bullying Questionnaire, which includes items about liking playtime, being alone at playtime, the number of good friends a child has, and their perceptions of their own popularity.	Results: victims of bullying overall report being left alone at playtimes and having fewer friends. Bullies and neutrals did not differ consistently.<br>Limits: vague definition and measurement of loneliness (i.e., not clearly distinguished from social exclusion).<br>Conclusions: bullying is a universal phenomenon with many negative correlates for victims and few for bullies. There are cultural differences in the way bullying is related to sex, age, and social support.
	Interpersonal Violence, Social connection and Loneliness	Check et al., 1985	Type of study: self-reports and behavioral.<br>Participants: 91 men.<br>Method: administration of the short survey version of the UCLA Loneliness Scale, the Acceptance of Interpersonal Violence Scale, the Hostility toward Women Scale. Men were also asked to punish (administering aversive noise) or reward the male/female confederate during a guessing task.	Results: lonely men expressed more hostility towards women and administered higher levels of aversive noise.<br>Limits: only male participants. Correlational study with no information on the direction of loneliness-hostility association. In the nearly 30 years since this publication, socio-cultural changes may have altered the phenomenon and reduced the current relevance of the results.<br>Conclusions: loneliness and social hostility might be related.
	Interpersonal Violence and Social connection	Zeng et al., 2021	Type of study: behavioral.<br>Participants: 101 violent and 171 non-violent offenders, 81 non-offending controls.<br>Method: emotion recognition test with morphed stimuli.	Results: compared to controls, both non-violent and violent offenders showed lower accuracy in recognizing emotions (especially disgust) but higher discrimination of happy faces. Non-violent offenders had lower fear recognition than violent offenders but higher anger recognition than controls.<br>Limits: the sample included only male East Asian adults and stimuli

				were limited to Caucasian faces (poor generalizability of findings and confounding race-effect).<br>Conclusions: facial recognition atypicalities are associated with aggressive behavior.
	Interpersonal Violence and Social connection	Nyline et al., 2018	Type of study: behavioral.<br>Participants: 35 male domestic violence offenders, 35 nonviolent men.<br>Method: facial emotion recognition test with morphed primary emotions: sadness, fear, disgust, anger, happiness, and surprise.	Results: offenders were less accurate in identifying sadness and fear, and identifying emotions of female faces compared to male faces. They were also less accurate in identifying realistic emotions (i.e., those at 40% and 60% emotional intensity).<br>Limits: control sample was older, had higher socioeconomic status, and more likely to be Caucasian than the offender sample. All facial stimuli were Caucasian.<br>Conclusions: inaccurate identification of the partner's emotion might obstruct social connection and facilitate domestic violence.
	Interpersonal Violence and Affective Touch	Strauss et al., 2019	Type of study: stimulation procedure, fMRI.<br>Participants:<br>(Experiment 1)13 female patients with interpersonal trauma associated with PTSD, 13 female healthy controls. (Experiment 2) 20 patients (19 women) with a history of interpersonal trauma, 20 healthy controls (19 women).<br>Method: five separate stroking conditions (stroking with hand vs stroking with brush), with varying amounts of CT fiber stimulation and visual control.<br>After each trial they were asked to assess pleasantness and intensity of stroking.<br>Questionnaire: PANAS, Touch deprivation questionnaire (Experiment 2)<br>fMRI. Stroking conditions differed in interpersonal stroking vs impersonal stroking and velocity (CT optimal vs CT suboptimal). Pleasantness assessment (self-reported).	Results: PTSD participants rated the two interpersonal conditions significantly more negative than controls.<br>Patients showed higher hippocampal activation for impersonal touch compared to the interpersonal touch; enhanced activation of the visual cortex (occipital lobe, region hOc2) for impersonal touch compared to the interpersonal touch. Both these effects were not observed in controls.<br>Limits: small, mainly female sample, medication could have impacted the BOLD data and hedonic perception. Experimenter was not blind to the participant's group membership. Stroking performed by hand might lack precision. Limited external validity for fMRI study.<br>Conclusions: Patients with interpersonal trauma associated with PTSD selectively dislike interpersonal touch. Patients rated interpersonal stroking touch as aversive. On a neural level, the observed touch aversion was related to enhanced hippocampal activation.<br>Interpersonal touch aversion in patients is independent from: the sex of the stroker, from whether the stroker can be seen or not and from whether stroking is performed to target C-tactile fibers or not.
	Interpersonal Violence and Virtual Reality	Seinfeld et al., 2018	Type of study: IVR, behavioral.<br>Participants: 20 male domestic violence offenders, 19 male controls.<br>Method: VR full body ownership illusion that makes offenders being in the body of a victim of domestic violence. Pre- and post-exposure emotion recognition test.	Results: offenders show lower ability to recognize fear in female faces compared to controls, with a bias towards classifying fearful faces as happy. After being embodied in a female victim, offenders improved their emotion recognition skills.<br>Limits: small only male sample.<br>Conclusions: IVR can modify perceptual processes and social

				skills, thought to underlie specific forms of violent behaviors.
	Interpersonal Violence, Affective Touch and Virtual Reality	Tremblay et al., 2016	Type of study: haptic technology, IVR.<br>Participants: 61 undergraduate university students (42 females) (mean age=19).<br>Method: sociodemographic questionnaire, anti-fat attitude questionnaire, Body Esteem Scale.<br>Procedure: participants were randomly assigned to one experimental condition with VH (virtual human) and haptic technology:<br>a) with female or male VH and (b) obese (BMI >30) or normal weight (BMI >18.5 and <24.9).<br>Participants were instructed to imagine that the VH was a stranger, and they were asked to give them an award. Using two haptic devices held by each hand, they were asked to give a hug to the VH.	Results: significant associations between body image satisfaction and anti-fat attitudes. Fear of fat was associated with lower satisfaction with body weight and appearance in women, while greater dislike of fat people was associated with lower satisfaction with weight and with belief of not being positively perceived by the others.<br>Female participants' greater weight satisfaction was associated with stronger touch intensity, whereas belief of positive perception from others was associated with longer touch duration.<br>Men's fat dislike was correlated with a weaker strength of touch.<br>Limits: small, mainly female sample, haptic devices are not a realistic replication of the hand (i.e., do not convey affective tactile feedback).<br>Conclusions: haptics can be used to assess individuals' interpersonal touch use, as an implicit measure of social attitudes.
